# Occurrence of D-amino acids in natural products

**DOI:** 10.1007/s13659-023-00412-0

**Published:** 2023-11-07

**Authors:** Daniel W. Armstrong, Alain Berthod

**Affiliations:** 1 Department of Chemistry and Biochemistry, University of Texas at Arlington, Arlington, TX 76019 USA; 2 Institut des Sciences Analytiques, CNRS, University of Lyon 1, 69100 Villeurbanne, France

**Keywords:** D-amino acid, Chirality, Biogenesis, Natural products

## Abstract

Since the identified standard genetic code contains 61 triplet codons of three bases for the 20 L-proteinogenic amino acids (AAs), no D-AA should be found in natural products. This is not what is observed in the living world. D-AAs are found in numerous natural compounds produced by bacteria, algae, fungi, or marine animals, and even vertebrates. A review of the literature indicated the existence of at least 132 peptide natural compounds in which D-AAs are an essential part of their structure. All compounds are listed, numbered and described herein. The two biosynthetic routes leading to the presence of D-AA in natural products are: non-ribosomal peptide synthesis (NRPS), and ribosomally synthesized and post-translationally modified peptide (RiPP) synthesis which are described. The methods used to identify the AA chirality within naturally occurring peptides are briefly discussed. The biological activity of an all-L synthetic peptide is most often completely different from that of the D-containing natural compounds. Analyzing the selected natural compounds showed that D-Ala, D-Val, D-Leu and D-Ser are the most commonly encountered D-AAs closely followed by the non-proteinogenic D-*allo-*Thr. D-Lys and D-Met were the least prevalent D-AAs in naturally occurring compounds.

**Graphical Abstract:**

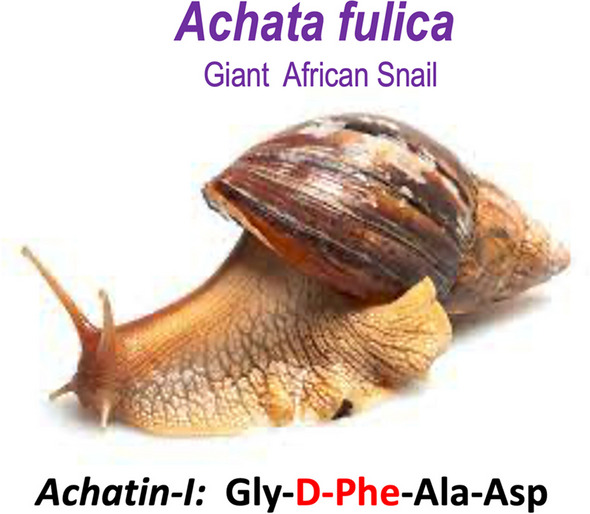

## Introduction

Amino acids (AA) are very simple chemicals containing both an amine and a carboxylic acid group [1]. In alpha-AAs, the amine and carboxylic acid groups are attached to the same carbon atom. Hydrogen is always the third substituent, hence, when the fourth and last substituent is not hydrogen (glycine), the α-AA is chiral. These α-AAs are the most important building blocks of living organisms given their ability to combine to form proteins. It is not yet well understood why the L-enantiomers are predominantly found in proteins that consist of no more than 19 L-α-AAs [2]. However, as early as 1894, Emil Fisher discovered enzymes, that he called Invertin and Emuslin, able to change the chirality of sugars [3]. Carrying on with amino acids, he identified their two possible D- and L-forms [4]. For years, D-AAs were believed to be present only in unicellular living forms such as viruses or bacteria with a variety of D-AAs found in peptides and antibiotics such as penicillin, the first antibiotic [5]. However, in 1981, D-Ala was unambiguously established in the sequence of dermorphin^1^**{*129*}**, a peptide extracted from the skin of the tree frog *Phyllomedusa* *sauvagine* [6]. Since then, numerous occurrences of D-AA have been found at different positions of bioactive peptides or proteins and in natural products [7]. Smaller amounts of free D-AAs are found in essentially all biological systems [8], sometimes playing vital roles and frequently being disease biomarkers [9].

This review focuses on D-AAs in natural products. What is their origin, how were they identified, and what is their function? D-aspartic acid was found in several tissues (teeth, bones, skin, lung, lens) of ageing living bodies, and D-serine was found, in addition to D-aspartic acid, in β-amyloid of Alzheimer patients [9, 10]. A wide variety of D-AAs can be found in the proteins of biological systems animal or vegetal, including mammal and humans as recently reviewed [8, 11]. These particular peptides or altered proteins will not be considered in this study. D-amino acids are also key building blocks in the biosynthesis of polyketide-nonribosomal hybrid peptides that found great interest in recent years [12–14]. To maintain a focused and manageable size for this review, these and other hybrid structures were not included. Rather, the large number of natural peptide compounds containing at least one D-AA is reviewed. These compounds were arbitrary sorted by their source: prokaryote bacteria and algae, eukaryote fungi, and multicellular invertebrate and vertebrate animals. Since the universal standard genetic code encodes only L-AAs, the presence of D-AAs in peptides and proteins is most frequently explained as the result of post-translational modifications or non-ribosomal biosynthesis. Non-proteinogenic AAs of the D- or L- configuration also must be considered in this context.

## Defining D-amino acids

D-AAs are the opposite (mirror image) enantiomeric forms of the 19 natural chiral proteinogenic α-AAs found in natural products. However, there are numerous non-proteinogenic amino acids that also have a stereogenic center. Table 1 lists a selection of unusual AAs encountered when searching for D-AAs in natural products. Several rare methylated or hydroxylated variants of these amino acids are not included in this table. These non-proteogenic AAs will be listed in the subsequent "natural compound" tables since they were commonly found associated with proteogenic D-AA containing peptides.

**Table 1:** Non proteogenic amino acids found in natural peptides sorted by increasing carbon number with L- or *S*- configuration shown.

Name	Structure	Formula	m.w	Code
Sarcosine^a^		C_3_H_7_NO_2_	89	Sar
β-Aminopropionic acid^a^		C_3_H_7_NO_2_	89	β-Ala
Selenocysteine L- but *R-*configuration		C_3_H_7_NO_2_Se	168	Sec
Cysteic acid L- but *R-*configuration		C_3_H_7_NO_5_S	169	CysA
2,3-Diaminopropionic acid		C_3_H_8_N_2_O_2_	104	Dpr
2,3-Dehydro-2-aminobutyric acid^b^		C_4_H_7_NO_2_	101	Dhb
Hydroxyaspartic acid^c^		C_4_H_7_NO_5_	149	OH-Asp
Hydroxyasparagine^c^		C_4_H_8_N_2_O_4_	148	OH-Asn
2-Aminobutyric acid		C_4_H_8_NO_2_	102	Abu
AlloThreonine^c^		C_4_H_9_NO_3_	119	allo-Thr
Homoserine		C_4_H_9_NO_3_	119	Hser
2,4-Diaminobutyric acid		C_4_H_10_N_2_O_2_	118	Dab
3-Hydroxyproline^c^		C_5_H_9_NO_3_	131	3Hyp
4-Hydroxyproline^c^		C_5_H_9_NO_3_	131	4Hyp
Norvaline		C_5_H_11_NO_2_	117	NorVal
Ornithine		C_5_H_12_N_2_O_2_	132	Orn
β-Homoproline^d^		C_6_H_11_NO_2_	129	β-Hpr
Pipecolic acid		C_6_H_11_NO_2_	129	Pip
Allo-isoleucine^c^		C_6_H_13_NO_2_	131	allo-Ile
Tertio-leucine		C_6_H_13_NO_2_	131	*t-*Leu
Enduracidine		C_7_H_11_N_4_O_2_	183	End
Citrulline		C_7_H_14_N_3_O_3_	188	Cit
Phenylglycine		C_8_H_9_NO_2_	151	PhGly
4-Hydroxyphenylglycine		C_8_H_9_NO_3_	167	Hpg
Phenylserine^c^		C_9_H_11_NO_3_	181	Phe-Ser
Kynurenine		C_10_H_11_N_2_O_3_	207	Kyn
Homotyrosine		C_10_H_14_NO_3_	196	Hty
Pyrrolysine^b,c^		C_12_H_2__1_N_3_O_3_	255	Pyl

^a^Achiral amino acids

^b^E or cis, and Z or trans possible isomerization

^c^Two asymmetric centers with 4 possible enantiomers: RR and SS; and RS and SR

^d^β-amino acid with the asymmetric center on β

## Identifying D-amino acids

Due to the identical physicochemical properties of enantiomers in isotropical environments, except for their chiroptical qualities, the presence of D-AAs is not easy to determine and can often be overlooked. Fisher was the pioneer especially interested in the chirality of molecules establishing the D and L nomenclature and testing his isolated compounds by artificially synthesizing them and using optical rotation to detect their chirality [3, 4]. Spectroscopic, chemical, and especially separation methods are used to characterize D-AAs [9].

### Spectroscopic methods

The spectroscopic methods using light for D-AA identification are optical rotation, called polarimetry, and circular dichroism spectroscopies. Raman optical activity is a new technique under development [15]. NMR spectroscopy uses very high frequency magnetic fields. ^1^H and ^13^C NMR spectra give structural information on the atom organization of the compound studied. Nuclear Overhauser Effect Spectroscopy (NOESY) is a powerful 2D NMR measurement giving information on the three-dimensional structure of biomolecules. Heteronuclear Single Quantum Coherence (HSQC) NMR gives correlations between carbons and protons that are separated by less than four bonds. Differences in NOESY or HSQC NMR assignments are seen between epimeric peptides differing in the chirality of amino acids. However, NMR methods are unable to determine the chirality of free AAs. Mass spectrometry analyses in vacuum detect charged compounds or fragments accelerated by electrical and magnetic fields. Fast-atom bombardment mass spectrometry (FAB-MS) uses a beam of neutral atomic gas (argon or xenon) bearing a high kinetic energy for the soft ionization of relatively large nonvolatile molecules with molecular weight up to 5000–6000 daltons and creation of charged fragments. Modern MS equipment can reach a m/z precision as low as 0.0001 Dalton allowing differentiation between a CH_2_ methylene group, m/z = 14.0156 Da, and a nitrogen atom, m/z = 14.0031. This was not possible with FAB-MS instruments but L- and D-enantiomers still cannot be differentiated by MS as they have the same exact mass.

The oldest and still very commonly used spectroscopic methods include polarimetry and circular dichroism methods [9]. Polarimetry measures the degree of rotation of a plane-polarized monochromatic light passing through a sample. Circular dichroism measures the absorption of left- and right-circularly polarized light of different wavelengths by a sample and displays the difference which is not nil for a chiral compound [9]. Polarimetry being linked to the solute refractive index is not very sensitive while circular dichroism can be somewhat more sensitive for solutes containing chromophores.

It was found that the optical properties of chiral molecules could be enhanced by orders of magnitude when adsorbed onto specific surfaces. Surface-enhanced polarimetry, circular dichroism and Raman optical activity methods using plasmonic nanostructures have potential in detecting smaller amounts of chiral molecules, but still are under developments [9].

### Chemical methods

Chemical methods include Edman degradation of the peptide chain, Marfey analysis, and chemical synthesis of the identified compounds for a direct comparison of their biological effects. The classic Edman degradation process involves reacting the N-terminal amino acid with phenylisothiocyanate, cleaving it with trifluoroacetic acid, and converting the resulting thiazolinone in aqueous acid to a stable phenylthiohydantoin (PTH) amino acid which is identified. The process is repeated for the next amino acid and the PTH derivatives, that have a strong UV absorption, are analyzed by HPLC. Edman degradation gives the amino acid sequence of a protein or peptide but cannot distinguish between D and L-forms unless a chiral column is used for the HPLC analysis [16].

The Marfey method is more specifically designed to identify the amino acid chirality. Its first step is to completely degrade the peptide in 6N HCl media. The second step consists in reacting the hydrolysate with Nα-(2,4-dinitro-5-fluorophenyl)-L-alaninamide (L-FDAA) or with its enantiomer D-FDAA to form UV absorbing diastereoisomers that can be separated by a classical C18 column and the peak position are compared with those of known FDAA derivatives of amino acid standards [17]. However, the strong acid hydrolysis step can partially racemize each chiral AA, and convert asparagine and glutamine to aspartic acid and glutamic acid, respectively, among other undesired effects.

Synthesizing amino acids by classical organic chemistry produces racemic mixtures, however pure D-AAs can be obtained by several different means. Feeding living organisms with a racemic amino acid mixture will leave a residue of D-AAs since only the L-forms are consumed by the organisms. Once the amino acid sequence of a natural product is established, this sequence is reproduced using L-AAs and comparing the properties of the synthesized product with those of the natural one. The D-Ala in Dermorphin** {*129*}** was identified by this method. Dermorphin has an analgesic potency two to three orders of magnitude higher than morphine. The synthetic all-L dermorphin lacked any analgesic activity [6].

### Separation methods

Separation methods are mainly chromatographic methods that separate compounds owing to their different affinities toward a stationary phase when they are carried by a mobile phase. Preparative liquid chromatography on low pressure columns usually is first used to isolate and purify the natural compounds. Thin layer chromatography (TLC), gas chromatography (GC), and high performance liquid chromatography (HPLC) were used to characterize the natural products or their amino acid constituents. The oldest approach to separate enantiomers is to derivatize them with an enantiomerically pure reagent prior to the chromatographic analysis. This is the principle of the Marfey method [17]. Classical HPLC, TLC, or GC for volatile derivatized compounds are able to separate the diastereoisomers obtained [9, 18]. In modern chiral studies, the derivatization approach has been supplanted by the use of chiral stationary phases (CSPs) which directly separate native molecules, hence avoiding several sample preparation steps.

Since enantiomers have identical properties in isotropic media, a chiral selector is needed to introduce some anisotropy and to allow for enantiomer differentiation. The chiral selector can make two transient diastereoisomers with both enantiomers. Once separated by the chromatographic process, the two enantiomers can be recovered independently. In chromatography, the chiral selector could be added to the mobile phase or attached to the stationary phase. Since chiral mobile phases need a continuous supply of expensive and pure chiral selector that must not disturb the detection method, CSPs are, by far, the method used in chromatography. Now, specific CSPs can routinely separate amino acid enantiomers that can be detected by MS or a variety of other detectors [19]. CSPs are more expensive chromatographic columns than classical columns. However, since the chiral selector is attached to the stationary phase, when used properly, CSP-containing columns can be used for hundreds or even thousands of enantiomer separations making the cost per separation insignificant [9]. The functionalized-carbohydrate CSPs based on bonded derivatized cellulose or amylose sugars cannot separate underivatized AAs or small peptides [19]. The macrocyclic glycopeptides based CSPs make chiral columns that are especially efficient in separating both native and N-blocked amino acids [20]. Recently introduced superficially porous particles bonded with chiral selectors allowed to obtain enantiomer separation in a very short time using minute amount of mobile phases [8, 9, 18, 19]. Associating achiral and chiral columns in 2D-HPLC greatly extended the capacity of the chromatographic methods allowing detecting trace amounts of chiral compounds [9, 18].

Peptide epimers that differ in the chirality of a single amino acid within the peptide chain are not enantiomers. They can be separated by achiral columns as demonstrated by the recent analysis of β-amyloids implicated in Alzheimer's disease [20]. However, CSPs [21] and especially macrocyclic glycopeptide bonded CSPs are particularly efficient in separating a wide variety of peptide epimers with minimum sample preparation [22, 23].

Capillary electrophoresis (CE) is a "micro-separation" method. However CE is not a chromatographic method and it separates compounds by their size to charge ratios using an electrical field created applying a high voltage in a capillary tube filled by the appropriate electrolyte. The CE technique requires that a chiral mobile-phase additive is dissolved in the running buffer, in order to separate any enantiomers, and especially D and L-AAs [9, 19]. CE provides high separation efficiency in relatively short electromigration times. The drawbacks are a mediocre reproducibility and sensitivity, and absence of any preparative capability.

## Origin/biosynthesis of D-amino acids in natural products

Feeding cultures of *Penicillium* *chrysogenium* with ^14^C marked D or L-Valine, Arnstein and Margreiter obtained penicillin **{*28*}** containing ^14^C D-Valine only when the mycelium was fed with L-Valine [24]. They demonstrated that only L-AAs were processed by the mycelium and that the antibiotic synthesis necessarily involved racemase or epimerase enzymes. The standard genetic code contains six different codons of three DNA bases for L-Arg, L-Leu, and L-Ser, four codons of three bases for L-Ala, L-Gly, L-Pro, L-Thr, and L-Val, three codons, all starting with AU: AUA, AUC, and AUG for L-Ile, two codons for L-Asn, L-Asp, L-Cys, L-Gln, L-Glu, L-His, L-Lys, L-Phe, and L-Tyr, with the single codon, AUG, encoding L-Met, and UGG encoding L-Trp. This genetic code is almost universal for all known living organisms animal or vegetal. However, so far, there is no identified codon for D-AAs. Hence, the synthesis of D-containing peptides or proteins cannot come directly from the translation of DNA nucleotide sequences in ribosomal peptide synthesis. Non-ribosomal peptide synthesis (NRPS) is the main biosynthetic approach that produces the unique structural features observed in natural products containing D-AAs [25].

**Fig. 1:**
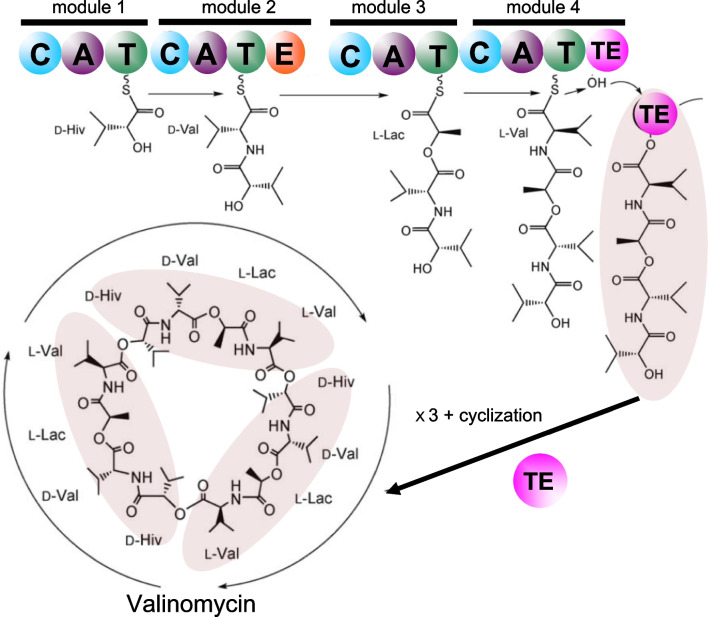
Simplified scheme of the valinomycin **{*43*}** biosynthesis by the NRPS subprotein of the bacteria *Streptomyces* *tsusimaensis* that was dissected into four modules containing a total number of fourteen domains (colored circles): *C* condensation, *A* adenylation, *T* thiolation, *E* epimerization, and *TE* C-terminal iterative domain. *D-Hiv* D-α-hydroxy isovaleric acid, *L-Lac* L-lactic acid. Adapted from [27]

Nonribosomally assembled peptides can be altered by subsequent chemical peptide synthesis or enzyme catalysis in a connected ballet between multi-domain NRPS proteins and polyketide synthases. NRPS enzymes are the largest known enzymes with molecular weights passing 2.3 MDa or more than 21,000 residues [26]. The NRPS structure was found to be made by successive modules, each containing a condensation domain, C, an adenylation domain, A, and thiolation domain, T. As an example, the production of valinomycin **{*43*}** by the bacteria *Streptomyces* *tsusimaensis* was fully described [27]. Four NRPS modules were identified to incorporate successively D-α-hydroxy isovaleric acid (Hiv), L-valine, L-lactic acid, and another L-valine. An epimerase domain, E, and an iterative terminal domain, TE, were also identified (Fig. 1). The E domain converts the first L-valine into D-valine, and the TE domain cleaves the tetrapeptide and catalyzes a head-to-tail cyclization to give valinomycin **{*43*}** after coupling three tetrapeptides. Advanced modern bioinformatics deciphered the *Streptomyces* *tsusimaensis* gene responsible for the NRPS valinomycin **{*43*}** production. This gene of 39,266 base pairs (bp) contains eighteen open reading frames (ORN) producing different proteins. From these 18 ORNs, the valinomycin (vlm) cluster was identified as ORN16 and ORN17. ORN16 was called vlm1 of 10,286 bps between bp 19,526 and bp 29,812, and ORN17 was called vlm2 of 7967 bps between bp 29,835 and 37,802 [27]. Both ORNs contained two modules making the four modules responsible for the valinomycin **{*43*}** production (Fig. 1). This example shows that racemase, epimerase or isomerase enzymes encoded in the NRPS processes are responsible for the occurrence of D-AAs in natural products as obtained from their L-counterparts.

The NRPS route is not the only natural process able to produce non-proteogenic and D-AA containing compounds. In the ribosomally-synthesized and post-translationally modified peptide (RiPP) synthesis route, organisms can synthesize compounds classically: normal codons for L-AAs are present in the mRNA at the position where the D-residue is found in the studied peptide. The L-AA is processed by a post-translational modification involving peptidyl-amino acyl L-D isomerization [8, 10]. Several mechanisms are possible. Simple epimerases just change a particular L-amino acid into its D-form using a deprotonation-protonation mechanism. The chiral AA hydrogen of a particular AA is removed to form an intermediate flat carbanion. The proton is reintroduced to the opposite side changing the chirality of the AA [28]. Hydroxylases or methylases are other enzymes that post-translationally modify L-AAs creating non-proteogenic AAs by converting the initial L-AA form into a D-form with hydroxyl or methyl groups added, respectively [29]. Also, specific enzymes are able to perform posttranslational conversion of a L-AA into another D-AA. For example, a lantibiotic synthetase found in *Lactococcus* *lactis* is able to change L-serine into D-alanine removing the hydroxyl group and changing the chirality in the production of the powerful lantibiotic 3147 **{*21*}** [30]. Replacing D-alanine by L-alanine in lantibiotic 3147 **{*21*}** reduced its bioactivity by 94% demonstrating that the type of AA and its chirality are both critical [30].

## Occurence of D-amino acids in natural compounds of various origins

### Prokaryotes

Prokaryotes are single-cell organisms lacking a nucleus. The domain of bacteria is of particular interest. They are also the first and most prevalent source of D-AA containing natural products. D-AAs were found in antibiotics, biosurfactants, toxins and siderophores produced by bacteria. All of these compounds facilitate bacterial life and survival. Antibiotics and toxins can limit or help eliminate competing bacteria or predator organisms. Biosurfactants facilitate bacterial spread in the medium or host. Iron is a critical element for bacterial development. Siderophores are very powerful iron-complexing compounds that selectively capture iron, releasing it specifically to the siderophore-producing strain. Table 2 lists D-AA containing compounds produced by prokaryotes, mainly bacteria [31–97]. The antibiotics are numbered and listed alphabetically with the bracketed number also used within this text after the compound name to facilitate reading. Compound names and numbers in the tables are followed by the name of the associated prokaryote, the structural formula, and molecular weight. The number of amino acids is given along with the number and proportion of D-AAs in each peptide. Also, the type of D-AAs and non-proteinogenic AAs are given in Table 2. The non-proteinogenic amino acids are listed using the Table 1 codes and adding the D- prefix if the D-form was found (Table 2).

**Table 2:** D-amino acids found in natural compounds coming from prokaryotic organisms (non-photosynthetic bacteria)

#	Name		Origin	Formula	m.w	Number of		%D/total	D-AAs	Non-prot. AAs	Refs.
						AAs	D-AAs				
*Antibiotics*											
***1***	A54145 A		*Streptomyces* *fradiae*	C_72_H_109_N_17_O_27_	1644	14	3	21.4	D-Glu, D-Lys, D-Asn	OH-Asn, N-Me-Gly, OMe-Asp	[31]
***2***	Actinomycin D		*Streptomyces* *antibioticus*	C_62_H_86_N_12_O_16_	1255	8	2	25.0	D-Val	Sar	[32]
***3***	Arylomycin A		*Streptomyces* *sp* *Tu* *6075*	C_42_H_60_N_6_O_11_	825	6	2	33.3	D-Ala	D-Me-Ser	[33]
***4***	Bacillomycin D		*Bacillus* *subtilis*	C_48_H_74_N_10_O_15_	1030	7	3	42.9	D-Asn, D-Ser, D-Tyr	–	[34]
***5***	Bacitracin A		*Bacillus* *subtilis*	C_66_H_103_N_17_O_16_S	1421	11	4	36.4	D-Asp, D-Glu, D-Phe	D-Orn	[35]
***6***	Bacitracin A1		*Bacillus* *licheniformis*	C_66_H_103_N_17_O_16_S	1422	12	4	33.3	D-Glu, D-Asp, D-Phe,	D-Orn	[36]
***7***	Bottromycin A2		*Streptomyces* *bottropensis*	C_42_H_62_N_8_O_7_S	822	7	1	14.3	–	D-methoxy-β-Ala-thiazole, Me-Pro, *t-*Leu	[37]
***8***	BT peptide		*Brevibacillus* *texasporus*	C_81_H_146_N_16_O_15_	1583	12	4	33.3	D-Leu, D-Lys, D-Tyr	D-Orn	[38]
***9***	Calcium dependant antibiotic		*Streptomyces* *coelicolor*	C_66_H_79_N_14_O_29_P	1562	11	3	27.3	D-Trp	D-HPG	[39]
***10***	Coistin		*Bacillus* *polymyxa*	C_54_H_102_N_16_O_12_	1166	10	1	10.0	D-Leu	Dab	[40]
***11***	Daptomycin		*Streptomyces* *roseosporus*	C_74_H_101_N_17_O_26_	1618	14	3	21.4	D-Ala, D-Asn	Orn, Me-Glu, Kyn	[41]
***12***	Daptomycin		*Streptomyces* *roseosporus*	C_72_H_101_N_17_O_26_	1620	13	3	23.1	D-Ala, D-Asn, D-Ser	Orn, Me-Glu	[42]
***13***	Daptomycin		*Streptomyces* *roseosporus*	C_72_H_101_N_17_O_27_	1620	13	3	23.1	D-Ala, D-Ser, D-Asn	Orn, Me-Glu	[43]
***14***	Empedopeptin		*Empedobacter*	C_49_H_79_N_11_O_19_	1125	8	4	50.0	D-Pro, D-Ser	D-β-OH-Ser	[44]
***15***	Enduracidin A		*Streptomyces* *fungicidus*	C_107_H_138_Cl_2_N_26_O_31_	2355	17	7	41.2	D-Ala, D-Ser	D-Orn, D-allo-Thr, Cit, D-End, D-Hpg	[45]
***16***	Fusaricidin C		*Paenibacillus* *polymyxa*	C_44_H_75_N_10_O_12_	935	7	4	57.1	D-Ala, D-Asn, D-Val	D-allo-Thr	[46]
***17***	Gramicidin		*Bacillus* *brevis*	C_97_H_139_N_19_O_17_	1842	15	6	40.0	D-Leu, D-Val	N-formyl-Val	[47]
***18***	Graticin		*Bacillus* *brevis*	C_78_H_110_N_14_O_14_	1467	12	4	33.3	D-Phe, D-Tyr	Orn	[48]
***19***	Iturin		*Bacillus* *subtilis*	C_47_H_72_N_12_O_14_	1028	7	3	42.9	D-Asn, D-Tyr	–	[49]
***20***	Jessenipeptin		*Pseudomonas* *sp.* *QS1027*	C_91_H_148_N_20_O_24_	1906	19	13	68.4	D-Ala, D-Pro, D-Val, D-Ile, D-Ser, D-Leu	Dhb, D-allo-Thr, Dab	[50]
***21***	Lacticin 3147A2		*Lactococcus* *lactis*	C_127_H_207_N_34_O_34_S_3_	2847	29	2	6.9	D-Ala	Abu, Dhb	[51]
***22***	Lysobactin		*Lysobacter* *sp.*	C_58_H_97_N_15_O_17_	1277	11	3	27.3	D-Leu, D-Arg	Ph-Ser, D-allo-Thr, OH-Asn	[52]
***23***	Massetolide B		*Pseudomonas* *sp.*	C_56_H_99_N_9_O_9_	1154	9	4	44.4	D-Glu, D-Ser, D-Val	D-allo-Thr	[53]
***24***	Mattacin		*Paenibacillus* *kobensis*	C_51_H_97_N_16_O_14_	1157	10	1	10.0	D-Leu	Dab	[54]
***25***	Milkisin C		*Pseudomonas* *fluorescens* *UCMA17988*	C_67_H_132_N_12_O_32_	1408	11	7	63.6	D-Asp, D-Glu, D-Ile, D-Leu, D-Ser, D-Thr		[55]
***26***	Mycobacillin		*Bacillus* *subtilis*	C_65_H_85_N_13_O_30_	1527	13	6	46.2	D-Asp, D-Glu	–	[56]
***27***	Neoviridogrisein I		*Streptomyces* *P8648*	C_45_H_64_N_8_O_10_	876	8	2	25.0	D-Leu, D-Pro	Abu, N-Me-Phe-gly, [3-hydroxy picolinic ac	[57]
***28***	Penicillin N		*Penicillium* *chrysogenum*	C_14_H_20_N_3_O_6_S	358	2	1	50.0	D-Glu		[58]
***29***	Plusbacin A		*Pseudomonas* *sp.*	C_49_H_83_N_11_O_21_	1161	8	4	50.0	D-Ala, D-Ser	Oh-Pro, D-OH-Asp. D-allo-Thr	[59]
***30***	Polymyxin B1		*Bacillus* *polymyxa*	C_56_H_98_N_16_O_13_	1202	10	1	10.0	D-Phe	Dab	[60]
***31***	Polymyxin B2		*Bacillus* *polymyxa*	C_55_H_96_N_16_O_13_	1189	10	1	10.0	D-Phe	Dab	[61]
***32***	Pristinamycin (Streptogramin B)		*Streptomyces* *virginiae*	C_71_H_84_N_10_O_17_	1349	7	1	14.3	D-Ala	–	[62]
***33***	Quinomycin C		*Streptomyces* *echinatus*	C_55_H_72_N_12_O_12_S_2_	1156	8	2	25.0	D-Ser	Me-Cys, diMe-allo-Ile	[63]
***34***	Ramoplanin A2		*Actinoplanes*	C_106_H_170_ClN_21_O_30_	2254	17	7	41.2	D-Ala	D-Orn, D-allo-Thr, D-Hpg	[64]
***35***	Ristocetin A		*Norcadia* *lurida*	C_95_H_110_N_8_O_44_	2066	7	2	28.6	D-Ala	D-MePhe	[65]
***36***	Syringomycin A1		*Pseudomonas* *syringae*	C_51_H_8_Cl_1_N_14_O_18_	1197	9	2	22.2	D-Ser	Dab, Dhb, OH-Asp	[66]
***37***	Syringopeptin 22		*Pseudomonas* *syringae*	C_100_H_179_N_24_O_27_	2147	22	16	72.7	D-Ala, D-Pro, D-Ser, D-Val	D-Dab, Z-Dhb, D-allo-Thr	[67]
***38***	Syringopeptin 25A		*Pseudomonas* *syringae*	C_113_H_183_N_27_O_30_	2398	25	15	60.0	D-Ala, D-Leu, D-Ser, D-Val	Dab, Dhb, D-allo-Thr, D-Dab	[68]
***39***	Teicoplanin A2		*Actinoplanes* *teichomyceticus*	C_88_H_97_Cl_2_N_9_O_33_	1878	7	3	42.9		D-Hpg, Cl-Tyr	[69]
***40***	Tolaasin B		*Pseudomonas* *tolaasli*	C_93_H_161_N_21_O_25_	1971	18	10	55.6	D-Pro, D-Val, D-Thr	D-Hser, D-Dab, D-allo-Thr	[70]
***41***	Triostin A		*Streptomyces* *sp.*	C_50_H_62_N_12_O_12_S_2_	1086	8	2	25.0	D-Ser	Me-Cys, Me-Val	[71]
***42***	Tyrocidine A		*Brevibacillus* *brevis*	C_66_H_87_N_13_O_13_	1270	10	2	20.0	D-Phe	Orn	[72]
***43***	Valinomycin		*Streptomyces* *tsusimaensis*	C_54_H_90_N_6_O_18_	1111	6	3	50.0	D-Val		[69]
***44***	Vancomycin		*Streptomyces* *orientalis*	C_66_H_75_Cl_2_N_9_O_24_	1449	7	3	42.9		NMe-Leu, D-OH-Tyr, D-Hpg	[73]
***45***	Xenoamicin A		*Xenorhabdus* *doucetiae*	C_64_H_109_N_13_O_15_	1299	7	4	57.1	D-Leu, D-Val, D-Ala	D-allo-Thr	[74]
*Siderophores*											
***46***	Azotobactin		*Azobacter* *vinelandii*	C_44_H_59_N_13_O_23_	1138	9	3	33.3	D-Ser	D-Cit, D-OH-Orn,	[75]
***47***	Chrysobactin		*Erwinia* *chrysanthemi*	C_16_H_23_N_3_O_7_	369	2	1	50.0	D-Lys		[76]
***48***	Corrugatin		*Pseudomonas* *corrugata*	C_40_H_67_N_13_O_19_	1033	8	1	12.5	D-Ser	Dab, OH-Asp, OH-His	[77]
***49***	Marinobactin E		*Halomonas* *aquamarina*	C_44_H_77_N_9_O_16_	987	6	3	50.0	D-Ser	D-threo-β-OH-Asp, Dab, N–OH-Orn, N-Ac-Orn	[78]
***50***	Pseudobactin A		*Pseudomonas* *putida*	C_42_H_62_N_12_O_16_	990	7	3	42.9	–	D-OH-Asp, D-OH-Orn, D-allo-Thr	[79]
***51***	Pyoverdin 1		*Pseudomonas* *fluorescens*	C_55_H_83_N_17_O_22_	1334	8	3	37.5	D-Ser	D-allo-Thr, OH-Orn	[80]
***52***	Pyoverdin G173		*Pseudomonas* *fluorescens*	C_49_H_69_N_13_O_21_	1175	7	2	28.6	D-Asp, D-Ser	Orn,	[81]
***53***	Pyoverdine 2		*Pseudomonas* *putida*	C_58_H_86_N_18_O_24_	1418	10	3	30.0	D-Ser, D-Gln,	D-allo-Thr, Orn, Dab	[82]
***54***	Pyoverdine I		*Pseudomonas* *aeruginosa*	C_55_H_83_N_17_O_22_	1334	8	2	25.0	D-Ser	OH-Orn	[83]
***55***	Staphylopine		*Staphylococcus* *aureus*	C_13_H_20_N_4_O_6_	328	3	1	33.3	D-His	Dab	[84]
*Cytotoxins (anticancer, herbicide, antifungal)*											
***56***	Amphisin		*Pseudomonas* DSS73	C_66_H_114_N_12_O_20_	1395	11	7	63.6	D-Asp, D-Gln, D-Leu, D-Ser	D-allo-Thr	[85]
***57***	Burkholdac A		*Burkholderia* *thilandensis*	C_22_H_35_N_3_O_6_S_3_	533	4	3	75.0	D-Cys, D-Met, D-Val		[86]
***58***	Cereulide		*Bacillus* *cereus*	C_57_H_96_N_6_O_18_	1153	6	3	50.0	D-Ala, D-Leu	–	[87]
***59***	Fengycin A		*Bacillus* *subtilis*	C_72_H_110_N_12_O_20_	1463	10	4	40.0	D-Ala, D-Tyr	D-Orn, D-allo-Thr	[88]
***60***	Fuscopeptin A		*Pseudomonas* *fuscovaginae*	C_86_H_139_N_21_O_22_	1818	19	14	73.7	D-Ala, D-Pro, D-Val	Z-Dhb, D-allo-Thr, D-Dab	[89]
***61***	Montanastatin		*Streptomyces* *anulatus*	C_36_H_60_N_4_O_12_	741	4	2	50.0	D-Val		[90]
***62***	Orphamide A		*Pseudomonas* *fluorescens* Pf-5	C_64_H_115_N_10_O_17_	1295	10	5	50.0	D-Glu, D-Ser	D-allo-Ile, D-allo-Thr	[91]
***63***	Pseudophomin A		*Pseudomonas* *fluorescens*	C_55_H_97_N_9_O_16_	1139	9	6	66.7	D-Glu, D-Ile, D-Leu, D-Ser	D-allo-Thr	[92]
***64***	Valinomycin		*Streptomyces* *anulatus*	C_54_H_90_N_6_O_18_	1111	6	3	50.0	D-Val		[69]
*Surfactants*											
***65***	Arthrofactin		*Arthrobacter* MIS38	C_64_H_11_N_11_O_20_	1354	11	7	63.6	D-Asp, D-Leu, D-Ser, D-Thr		[93]
***66***	Kurstakin		*bacillus* *Thuringiensis*	C_40_H_65_N_11_O_12_	891	7	2	28.6	D-Glu, D-Thr	–	[94]
***67***	Serrawettin W2		*Serratia* *marcescens* NS25	C_38_H_61_N_5_O_9_	731	5	2	40.0	D-Leu, D-Phe	–	[95]
***68***	Tensin		*Pseudomonas* *fluorescens*	C_67_H_115_N_12_O_20_	1407	11	7	63.6	D-Asp, D-Gln, D-Leu, D-Ser	D-allo-Thr	[96]
***69***	Viscosin		*Pseudomonas* *fluorescens*	C_54_H_97_N_9_O_17_	1125	9	4	44.4	D-Glu, D-Ser, D-Val	D-allo-Thr	[97]

**Fig. 2:**
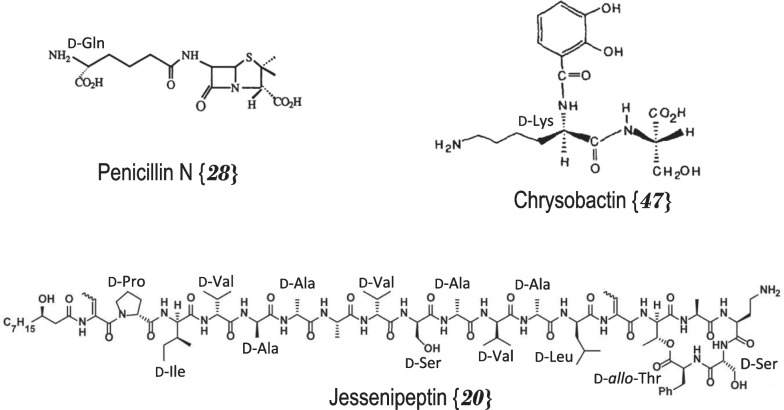
Structure of D-amino acid containing natural compounds produced by bacteria. The bracketed numbers refer to the Table 2 number codes along with additional information

The syringopeptin antibiotics **{*36*}*,*** **{*37*}** and **{*38*}** from the bacteria *Pseudomonas* *syringae* are among the larger peptides listed, containing up to 25 amino acids with up to 16 in the D-form [66–68]. Jessenipeptin **{*20*}** from *Pseudomonas* sp.QS1027 is another large antibiotic with 19 AAs, 13 of which are the D-antipodes (Fig. 2) [50]. The first and best-known penicillin **{*28*}** antibiotic from *Penicillium* *chysogenum* and the siderophore chrysobactin **{*47*}** from *Erwinia* *chrysanthemi* are made up of only two AAs, one being in its D-form: respectively D-glutamine [58] and D-lysine [76] (Fig. 2). There is no direct or obvious relationship between the total number of amino acids in a compound and the number of which are of the D-configuration although larger peptides could accommodate more D-AAs (Table 2). The phytotoxin fuscopeptin A **{*60*}** from *Pseudomonas* *fuscovaginae* consists of 19 AAs with 74% or 14 in the D-form (Fig. 2) [89]. The small cytotoxic burdolhac A **{*57*}** from *Burkholderia* *thilandensis* has a similar D-proportion with only four amino acids, three of which are the D-configuration [86]. On the other hand, the antibiotics daptomycin **{*11*}** from *Sterptomyces* *roseosporus* or mattacin **{*24*}** from *Paenibacillus* *kobensis* contain respectively 14 and 10 total AAs but only respectively three (21%) [36] and one (10%) [48] are in the D-form.

The small Staphylopine **{*55*}** produced by the MRSA *Staphylococcus* *aureus* is a metallophore that is extremely efficient in complexing zinc. This property explains its high pathogenicity since the host defense, called nutritional immunity, consists in restricting zinc availability critical to bacterial development [84]. Staphylopine **{*55*}** contains a D-histidine unit that acts as the metal chelating group.

Most of the listed antibiotics were synthesized by bacteria using a NRPS process. Bottromycins **{*7*}** are powerful heptapeptides produced by *Spectromyces* *bottropensis* that were extensively studied. It was found that they were produced following a RiPP pathway with a ribosomally produced core peptide that is post-translationally modified by tailoring enzymes [36]. Lacticin 3147A2 **{*21*}** is a larger antibiotic with 29 amino acids and only 2 D-AAs. It is synthesized by *Lactococcus* *lactis* also via a RiPP process [44]. Lacticin 3147A2 **{*21*}** is a lantibiotic compound, meaning that it contains the pseudo-amino acid lanthionine which is enzymatically created by connecting two alanines, or aminobutyric acid and alanine via a sulfur atom. Lacticin A2 **{*21*}** synergistically works with its other RiPP produced lacticitin 3142A1 compound inducing pore formation in bacterial walls [29, 30, 51]. Lantibiotics are much more powerful than antibiotics and offer hope in overcoming antibiotic resistant *Staphilococcus* *aureus* and other lethal bacteria [51].

### Photosynthetic microorganisms (prokaryote cyanobacteria and eukariote algae)

Prokaryotic cyanobacteria and eukaryotic algae contain pigments that allow them to perform photosynthesis producing oxygen associating them to the plant kingdom. Table 3 lists a selection of D-amino containing compounds produced by cyanobacteria or algae [98–107]. These relatively small compounds were sought for their biological activity as indicated in this table. Amphibactin **{*70*}** from the protobacterum *Vibrio* sp.R-10 is an amphiphilic siderophore that collects the rare iron ions present in the marine environment [98]. All the other listed marine natural products that contain D-AAs are cytotoxins (Table 3). Microcyctin **{*78*}** produced by the algae *Nostoc* *sp.152* is a particularly virulent hepatotoxin [104].

**Table 3:** Photosynthetic micro-organisms producing D-amino acid containing natural products: prokaryotic cyanobacteria and eukaryotic algae and plankton

#	Name	Origin	Biological effect	Formula	m.w	Number of		%D/total	D-AAs	Non-prot. AAs	Refs.
						AAs	D-AAs				
***70***	Amphibactin G	*γ-protobacterum* *vibrio* *sp.* *R10*	Siderophore	C_42_H_75_N_7_O_14_	901	4	2	50	D-Gln	D-Orn	[98]
***71***	Anabaenopeptin G	*Oscillatoria* *agardhii*	Carboxypeptidase inhibitor	C_45_H_68_N_10_O_9_	890	6	1	16.7	–	D-formyl-Lys, Hty, N-Me-Hty,	[99]
***72***	Ferintoic acid	*Mycrocystis* *aeruginosa*	Hepatotoxin	C_46_H_58_N_8_O_9_	866	6	1	16.7	–	N-Me-Ala, Hty, D-formyl-Lys	[100]
***73***	Kahalalide B	Alga *Bryopsis* *sp.*	Antibiotic	C_46_H_67_N_7_O_11_	893	7	4	57.1	D-Leu, D-Phe	–	[101]
***74***	Kahalalide F		Oncosis agent	C_75_H_124_N_14_O_16_	1477	13	7	53.8	D-Pro, D-Val	D-allo-Ile, Orn, D-allo-Thr, Dhb,	[101]
***75***	Kahalalide G		Antitumor	C_75_H_126_N_14_O_17_	1495	13	7	53.8	D-Pro, D-Val	4OH-Pro	[101]
***76***	Kahalalide H		Antitumor	C_55_H_82_N_8_O_16_	1111	8	4	50	D-Asp, D-Leu, D-Phe, D-Val	D-allo-Ile, Orn, D-allo-Thr, Dhb,	[101]
***77***	Kahalalide K		AIDS opportunistic infections	C_46_H_66_N_7_O_11_	891	6	3	50	D-Ala, D-Asn, D-Phe	4OH-Pro	[102]
***78***	Microcystin 1	*Alga* *Microcistis*	Hepatotoxin	C_49_H_75_N_10_O_12_	995	6	3	50	D-Ala, D-Glu	D-Me-Asp	[103]
***79***	Microcystin LR	*Nostoc* *sp.* *152*	Hepatotoxin	C_49_H_72_N_10_O_13_	1008	7	3	42.9	D-Asp, D-Ala, D-Glu	Me-Ser	[104]
***80***	Nodularin	Plankton *Nodularia* *spumigena*	Antibiotic	C_41_H_60_N_8_O_10_	824	5	2	40	D-Glu	Adda, D-Me-Asp	[105]
***81***	Oscillarin	*Oscillatoria* *agardhii*	Antithrombotic	C_34_H_44_N_6_O_5_	616	1	1	100	D-Phe		[106]
***82***	Peptide OA29	*Oscillatoria* *agardhii*	Hepatotoxin	C_48_H_74_N_13_O_12_	1024	7	3	42.9	D-Ala,	D-isoAsp, D-isoGlu	[107]

### Fungi

Fungi are eukaryotic multi-cellular organisms that are unable to directly synthesize essential nutrients. They are heterotrophic living entities that depend on other organisms for their sustenance. Fungi- produced peptides and depsipeptides that have been found to contain D-AAs are listed in Table 4 [108–119]. They have various biological effects, most helping the fungus to survive. The cytotoxic fungal compounds were found to be pharmacological interesting, having anticancer, antimalarial, or antibiotic properties (Table 4). Malformins **{*90–92*}**, a plant toxin produced by the fungus *Aspergillus* *niger*, was extensively studied, presenting numerous variations [115–118]. Studying the activity of artificially synthesized malmorfin compounds, it was demonstrated that the D-Cys and D-Leu amino acids found in the structure were critical for their biological activity (Fig. 3) [118]. D-His is rarely encountered in natural products. However, the ergot fungus *Verticilium* *kibiense* produces a polypeptide associating five L-Arg-D-His dipeptides [119]. This decapeptide was the sole natural compound reported to date in the literature that contained the rare D-histidine moiety.

**Table 4:** D-containing peptides and depsipeptides produced by fungi, either unicellular yeast or multicellular mushrooms

#	Name	Origin	Biological activity	Formula	m.w	Number of		%D total	D-AAs	Non-prot. AAs	Refs.
						AAs	D-AAs				
***83***	Apicidin D2	*Fusarium* *pallidoroseum*	Antimalarial	C_34_H_51_N_5_O_6_	625	4	1	25	–	D-Hpr	[108]
***84***	Beauverolide N	*Beauveria* *bassiana*	Insecticide	C_27_H_41_N_3_O_6_	503	3	1	33.3	D-Leu	–	[109]
***85***	Chlamydocyn	*Diheterospora* *chlamydosporia*	Antitumor	C_28_H_38_N_4_O_6_	526	4	1	25	D-Pro	2Me-Ala	[110]
***86***	Cyclosporin A	*Beauveria* *nivea*	Immuno suppresive agent	C_62_H_111_N_11_O_12_	1202	11	1	9.1	D-Ala	N-Me-Leu, Abu, Sar, Me-Val, N-Me-Gly	[111]
***87***	HC-toxin	*Cochliobolus* *carbonum*	Toxin	C_21_H_32_N_4_O_6_	436	4	2	50	D-Ala	2-amino-9,10-epoxi-8-oxodecanoic acid	[112]
***88***	HC-toxin B	*Cochliobolus* *carbonum*	Toxin	C_21_H_32_N_4_O_7_	452	4	2	50	D-Ala	2-amino-9,10-epoxi-8-oxodecanoic acid	[113]
***89***	Isariin	*Isaria* *Felina*	Insecticide	C_33_H_59_N_5_O_7_	637	5	1	20	D-leu	–	[114]
***90***	Malformin A2	*Aspergillus* *niger*	Plant toxin	C_22_H_37_N_5_O_5_S_2_	515	5	3	60	D-Cys, D-Leu	–	[115]
***91***	Malformin B	*Aspergillus* *niger*	Plant toxin	C_23_H_39_N_5_O_5_S_2_	529	5	3	60	D-Cys, D-Ile	–	[116]
***92***	Malformin C	*Aspergillus* *niger*	Plant toxin	C_23_H_39_N_5_O_5_S_2_	529	5	3	60	D-Cys, D-Leu	–	[117]
***93***	Allomalformin	artificially synthesized	Plant toxin	C_23_H_39_N_5_O_5_S_2_	529	5	3	60	D-Cys, D-Leu	–	[118]
***94***	Poly(Arg-His)5	*Verticilium* *kibiense*	Antibiotic	C_66_H_97_N_35_O_11_	1555	10	5	50	D-His	–	[119]

**Fig. 3:**
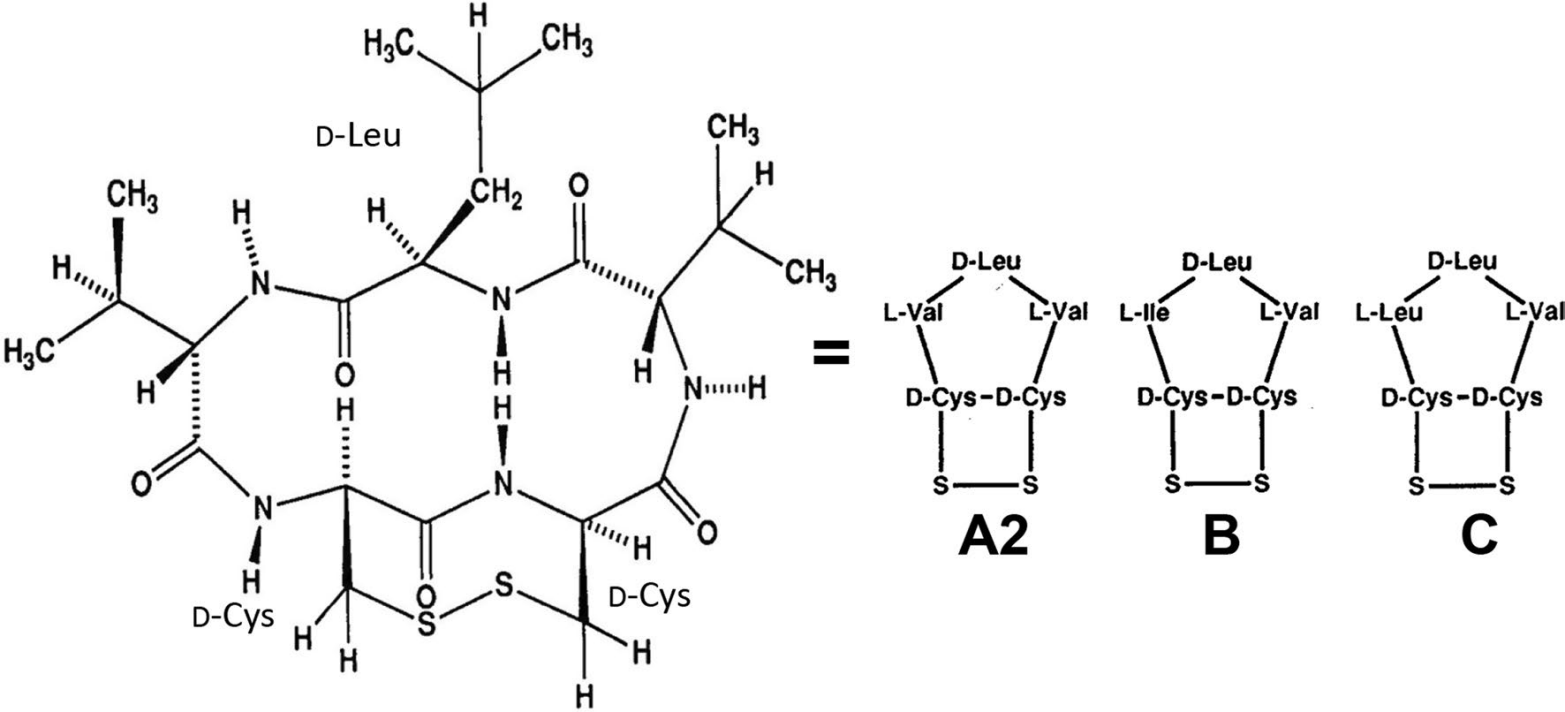
Structure of Malformin A2 **{*90*}**, B **{*91*}** and C **{*92*}** produced by the fungus *Aspergillus* *niger* showing the constant position of the three D-amino acids. The bracketed numbers refer to the Table 4 number codes along with additional information

### Multicellular organisms

Table 5 lists the D-AA containing natural compounds found in both invertebrate and vertebrate animals [101, 102, 120–150]

**Table 5:** D-amino acid containing natural products found in invertebrate and vertebrate animals

	Name	Origin	Animal	Biological activity	Formula	Number of		D-AAs	D/total	D-AAs	Non-prot. AAs	Ref
						m.w	AAs					
*Invertebrates*												
***95***	Kahalalide A	*Elysia* *rufescens*	Mollusk	AIDS opportunistic infections	C_46_H_67_N_7_O_11_	894	7	4	57.1%	D-Leu, D-Phe	–	[120]
***96***	Kahalalide B			Antitumor	C_45_H_63_N_7_O_11_	878	7	1	14.3%	D-Leu	–	[120]
***97***	Kahalalide C			Antitumor	C_47_H_63_N_9_O_10_	914	6	3	50%	D-Phe, D-Tyr	–	[120]
***98***	Kahalalide D			AIDS opportunistic infections	C_31_H_45_N_7_O_5_	595	3	1	33.3%	D-Trp	–	[101]
***99***	Kahalalide E			Herpes	C_45_H_69_N_7_O_8_	836	6	2	33.3%	D-Ala	–	[101]
***100***	Kahalalide F			Anticancer	C_75_H_124_N_14_O_16_	1477	13	5	38.5%	D-Ile, D-Pro, D-Val	Z-Dhb, D-allo-Ile, D-allo-Thr	[101]
***101***	Kahalalide J			Anticancer	C_61_H_94_N_10_O_17_	1239	9	4	44.4%	D-Asp, D-Leu, D-Phe, D-Val	4OH-Pro	[101]
***102***	Kahalalide K			AIDS opportunistic infections	C_46_H_66_N_7_O_11_	892	6	3	50%	D-Ala, D-Asn, D-Phe	–	[102]
***103***	Kahalalide R	*Elysia* *gandifolia*	Mollusk	Anticancer	C_75_H_124_N_14_O_16_	1477	13	5	50%	D-Pro, D-Val	D-allo-Thr, D-allo-Ile, Dhb	[121]
***104***	Kahalalide V	*Elysia* *rufescens*	Mollusk	Antitumor	C_31_H_47_N_7_O_6_	614	3	1	33.3%	D-Trp	–	[122]
***105***	Onchidin B	*Onchidium* *sp.*	Mollusk	Antitumor	C_62_H_96_N_4_O_16_	1152	4	1	25%	D-Pro	Me-Val	[123]
***106***	Achatin I	*snail* *Achata* *fulica*	Snail	Neuropeptide	C_18_H_40_N_4_O_6_	408	4	1	25%	D-Phe		[124]
***107***	Barangamide B	*Theonella* *swinhoe*	Sponge	Immuno suppressant	C_53_H_95_N_11_O_12_	1077	11	4	36.4%	D-Leu,	D-allo-Ile, NMe-Val, βAla, D-NMe-Val	[125]
***108***	Callipeltin A	*Callipelta* *sp.*	Sponge	Antitumor	C_68_H_116_N_18_O_20_	1506	9	4	44.4%	D-Arg	DiMe-Gln, Me-Gln, Me-Ala	[126]
***109***	Callipeltin E	*Latrunculia* *sp.*	Sponge	Antifungal antitumor	C_36_H_60_N_10_O_11_	808	5	2	40%	D-Arg	D-allo-Thr	[127]
***110***	Callipeltin K	*Latrunculia* *sp.*	Sponge	Antifungal antitumor	C_67_H_116_N_18_O_21_	1508	9	5	55.6%	D-Arg	D-Me-Gln, D-allo-Thr, N-Me-Gln, D-OMe-Tyr, N-Me-Ala	[128]
***111***	Cupolamide	*Theonella* *cupola*	Sponge	Cytotoxic	C_42_H_67_N_11_O_14_SNa	1004	7	3	42.9%	D-Leu, D-Ser	D-homoArg, Dab	[129]
***112***	Cyclotheonamide A	*Theonella* *swinhoe*	Sponge	Anticoagulant	C_36_H_45_N_9_O_8_	731	5	1	20%	D-Phe	α-ketoArg, VinylTyr	[130]
***113***	Discodermin E	*Discoderma* *kiiensis*	Sponge	Antibiotic	C_76_H_117_N_20_O_23_S	1709	14	5	35.7%	D-Ala, D-Leu, D-Cys,	D-t-Leu, D-Kyn, NMe-Gln, NMe-Gly	[131]
***114***	Halicylindramide A	*Halichondria* *cylindrata*	Sponge	Cytotoxic	C_78_H_111_BrN_20_O_22_S	1791	14	5	35.7%	D-Val, D-Trp, D-Phe	Br-Phe, N-Me-Gln, Sar, D-formyl-Ala, D-CysA	[132]
***115***	Keramamide E	*Theonella* *sp.*	Sponge	Antitumor	C_53_H_75_BrN_10_O_12_	1122	7	3	42.9%	D-Leu, D-Ile	D-allo-Ile, Orn, nor-Val	[133]
***116***	Koshikamide A2	*Theonella* *sp.*	Sponge	Cytotoxic	C_72_H_112_N_16_O_16_	1457	11	2	18.2%	D-Phe	NMe-Leu, NMe-Val, NMe-Asn, NMe-Ile	[134]
***117***	Microsclerodermin J	*Microscleroderma* *herdmani*	Sponge	Antifungal	C_46_H_60_N_8_O_12_	916	6	2	33.3%	D-Trp	N-Me-Gly, D-OH-Asp	[135]
***118***	Microspinosamide	*Sidonops* *microspinosa*	Sponge	Anti HIV	C_75_H_109_BrN_18_O_22_S	1725	13	6	46.2%	D-Ala, D-Asp, D-Cys, D-Trp, D-Val	D-*t*-Leu, N-Me-Glu	[136]
***119***	Motuporin	*Theonella* *swinhoei*	Sponge	Protein phosphatase inhibitor	C_40_H_57_N_5_O_10_	768	5	3	60%	D-Glu	D-Me-Asp	[137]
***120***	Mozamide A	*Theonella* *swinhoe*	Sponge	Unknown	C_45_H_64_N_8_O_9_	860	6	1	16.7%	D-Val	allo-Ileu	[138]
***121***	Neamphamide A	*Neamphamius* *huxleyi*	Sponge	Anti HIV	C_75_H_125_N_21_O_23_	1687	12	4	33.3%	D-Arg, D-Thr, D-Asn, D-Pro	N-Me-Gly, OMe-Tyr,	[139]
***122***	Phoriospongin A	*Callyspongia* *bilamellata*	Sponge	Nematocide vermifuge	C_52_H_83_ClN_11_O_15_	1137	10	5	50.0%	D-Ala, D-Asn	D-Me-NorVal, formyl-Leu, D-Norval, D-allo-Thr	[140]
***123***	Polydiscamide A	*Discodermia* *sp.*	Sponge	Anticancer	C_75_H_109_BrN_19_O_2_S	1706	13	7	53.8%	D-Ala, D-Asn, D-Trp, D-Cys, D-Leu, D-Val	D*-t-*Leu*,* *Sar*	[141]
***124***	Polydiscamide B	*Ircinia* *sp.*	Sponge	Pain killer	C_75_H_109_BrN_18_O_21_S	1708	13	6	46.2%	D-Ala, D-Asp, D-Cys, D-Trp, D-Val	Br-Phe, Me-Ile, D-t-Leu, N-Me-Gln	[142]
***125***	Polytheonamide A	*theonella* *swinhoei*	Sponge	Cytotoxic	C_219_H_376_N_60_O_72_S	5029	48	18	37.5%	D-Ala, D-Ser, D-Thr, D-Leu, D-Asp	Me-Ile, D-OH-Asp, D-Me-Glu, D-OH-Val	[143]
***126***	Pseudotheonamide A1	*Theonella* *swinhoei*	Sponge	Serine protease inhibitor	C_36_H_45_N_9_O_8_	731	5	1	20.0%	D-Phe	Dpr, k-Arg	[144]
***127***	Theonellapeptolide Ie	*Theonella* *swinhoe*	Sponge	Cytotoxic	C_70_H_126_N_13_O_16_	1404	13	5	38.5%	D-Leu	D-NMe-Leu, NMe-βAla, N-Me-Ile, βAla, D-allo-Ile, N-Me-Val, D-NMe-allo-Ile	[145]
***128***	Theonellapeptolide IIIe	*Lamellomorpha* *strongylata*	Sponge	Antitumor	C_71_H_127_N_13_O_16_	1417	12	4	33.3%	D-Leu, D-Val	D-Me-Leu, N-Me-Ala, N-Me-Ile, D-allo-Ile	[146]
*Vertebrates*												
***129***	Dermorphin	*Phyllomedusa* *bicolor*	Frog	Opioid	C_40_H_98_N_8_O_7_	802	7	1	14.3%	D-Ala	–	[147]
***130***	Dermenkephalin	*Phyllomedusa* *sauvagei*	Frog	Opioid	C_44_H_62_N_10_O_10_S_1_	955	7	1	14.3%	D-Met	–	[148]
***131***	Bombinin H4	*Bombina* *variegata*	Toad	Antimicrobial	C_91_H_175_N_22_O_22_	1927	20	1	5.0%	D-allo-Ile	–	[149]
***132***	Defensin DLP-2	*Ornithorynchus* *anatinus*	Platypus	Venom	C_216_H_321_N_62_O_59_S_9_	5013	42	1	2.4%	D-Met	–	[150]

*5.4.1.* *Invertebrates:* Most of the invertebrates that were found to produce D-AA containing compounds were marine mollusks, sponges and snails. The peptide and most often depsipeptide compounds found in invertebrates were relatively small with less than 15 amino acids and molecular weights lower than 1800 Da and were produced by NRPS processes. Kahalalides, depsipeptides from the herbivorous *Elysia* marine mollusk family and their algal diet *Bryopsis* *pennata* were extensively studied and presented wide structural variations and promising pharmacological properties. Kahalalide D **{*98*}** and V **{*104***} are small tripeptides each containing a D-Trp unit (Table 5). Kahalalide R **{*103*}** is a tridecapeptide containing five D-AAs. Kahalalides are cytotoxic and active against cancer cells or AIDS opportunistic infectious bacteria [101, 102, 120, 121]. All reported kahalalides were produced by NRPS processes [101].

An interesting exception in the making of invertebrate natural compounds is the largest compound in Table 5; polytheonamide A **{*125*}** with 48 amino acids wherein 18 are in the D-form (37.5%) with a total mass of 5029 Da [143]. Polytheonamide A **{*125*}** is produced by the sponge *Theonella* *swinhoei*. It was demonstrated that polytheonamide **{*125*}** is a RiPP produced compound: it was initially synthesized by the normal ribosome pathway with all proteinogenic L-AAs and then modified through numerous epimerisations. It contains 18 different D-AAs (Table 5). Also, specific N-methyltransferase reactions introduced several non-proteogenic D-amino acids [29, 151]. A single epimerase, named PoyD, generated all D-AA residues acting by protonation-deprotonation of the α-hydrogen of the L-AA [29]. This post-translationally modified compound has cytotoxic capabilities by creating membrane channels acting on cation circulation [143, 151].

**Fig. 4:**
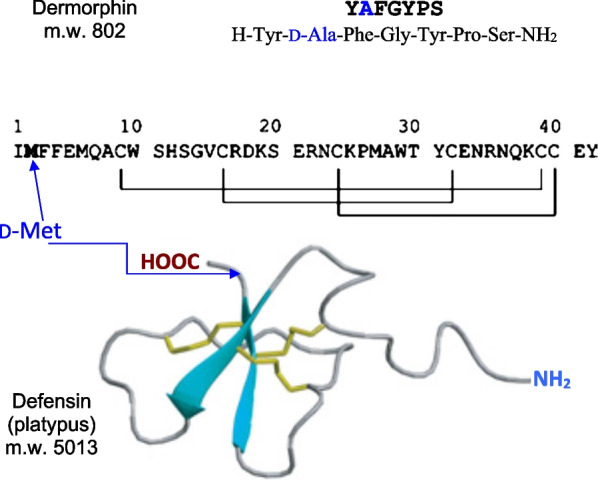
Top: the 7 amino acid sequence of dermorphin **{*129*}**, the first natural compound containing a D-amino acid found in vertebrates. Bottom: the 42 amino acid sequence of the platypus defensin **{*132*}** all L, except D-methionine in position 2. The calculated ribbon quaternary structure of the large peptide shows the disulfide connectivities in yellow and secondary-structural element attractions in blue [150]. The bracketed numbers refer to the Table 5 number codes along with additional information

*5.4.2.* *Vertebrates:* Since the discovery of D-alanine in dermorphin **{*129*}** produced by the frog *Phyllomedusa* *bicolor* [6] (Fig. 4), a few more natural compounds containing D-AAs were reported in vertebrates as listed in Table 5. Defensin DLP-2 **{*132*}** was found in the venom of the mammal *Ornithorynchus* *anatinus*, the platypus of Australia. Out of the 42 AAs found in this defensive venomous natural product only one had the D-configuration (Table 5 and Fig. 4). It is interesting to note that the synthesis of the all-L version of the venom has the same biological activity as the natural D-methionine containing, compounds. However, the natural and synthetic isomeric peptides showed significant differences in their liquid chromatography reversed-phase retention times [152]. An isomerase, glycoprotein of 52 kDa was found in frog skin secretion [151]. It could convert the L-form of the second amino acid residue into its D-form suggesting a RiPP synthesis of the observed D-AA containing compound **{*129*}** found in the frog [152].

## Outlook/overview of D-amino acid containing natural products

The selection of 132 D-containing natural products presented in this work consisted of 69 compounds found in eukaryotic bacteria (Table 2), 13 compounds found in marine eukaryotic cyanobacteria and prokaryotic algae (Table 3), 12 compounds found in fungi (Table 4), and 38 compounds found in invertebrate and vertebrate animals (Table 5). These 132 compounds contained a cumulated number of 1166 amino acids of which 442 had the D-configuration (37.9%).

**Fig. 5:**
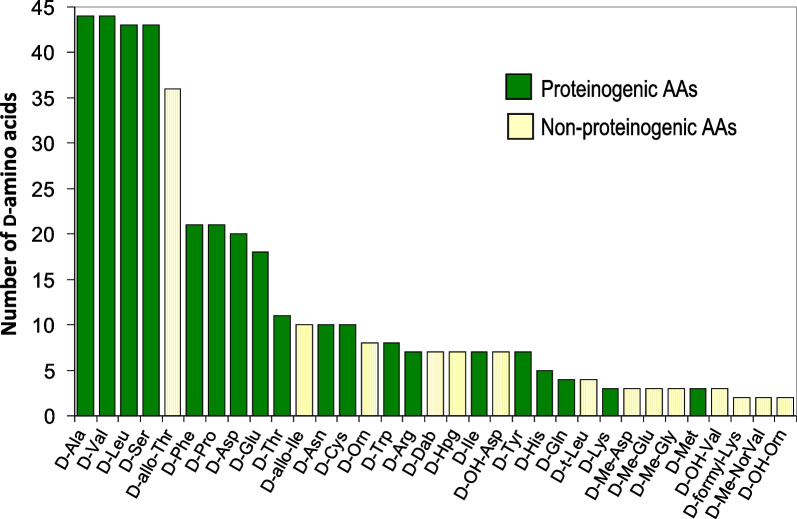
Occurrence of the D-amino acids founds in a set of 128 peptidic compounds. Total number of D-amino acids = 442. Green bars: proteinogenic amino acids; orange bars: non-proteinogenic amino acids. See Table 1 for AA codes and Tables 2, 3, 4, 5 for full data

Figure 5 presents the occurrence of D-AAs found in the analyzed set of 132 compounds. The non-proteinogenic amino acids are shown with light yellow bars. 329 proteinogenic D-AAs (green bars) covering the whole set of the 19 amino acids having a stereogenic carbons were counted. The two most encountered D-proteinogenic amino acids were D-Ala and D-Val closely followed by D-Leu and D-Ser. D-allo-threonine is next being the most encountered D-non-proteogenic amino acid of the 113 found (Tables 2, 3, 4, 5). It is pointed out that threonine has two asymmetric centers, hence four enantiomeric forms. L- and D-threonine have respectively the (S-R) and (R-S) configurations and L- and D-allo-threonine are the (S-S) and (R-R) enantiomers. A similar stereochemistry is found with isoleucine. All the proteinogenic L-AAs are of the S-configuration except for cysteine (and selenocysteine) in which the sulfur (or selenium) heavier atom alters the sequence making it of the R-configuration according to the Cahn-Ingold-Prelog rules [1, 2].

**Fig. 6:**
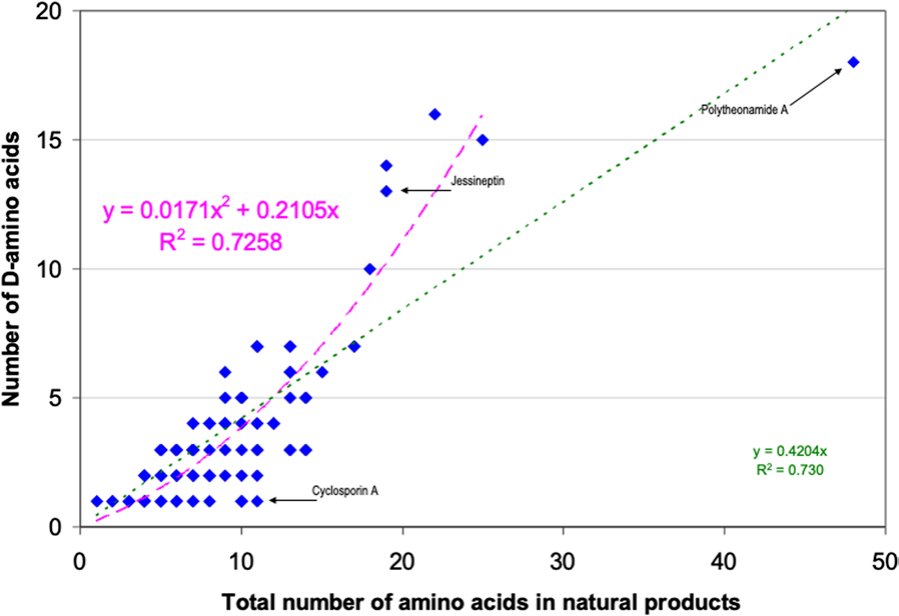
Comparing the number of D-amino acids found in a natural product with the total number of AA in the peptide. Several points correspond to more than one compound. Linear fit in green, quadratic fit excluding polytheonamide A ***125*** in purple. See text and Tables 2, 3, 4, 5 for full data

Sixteen D-non-proteinogenic AAs were not shown in Fig. 2 because they were encountered only in one compound. These D-non-proteinogenic AAs included D-citrulline found in the siderophore azobactin **{*46*}** (Table 2) or D-kynurenine found in discodermin E **{*113*}** (Table 5). Likely because there are a large variety of biologically modified AAs (Table 1), the number of encountered non-proteinogenic D-AAs (light yellow bars in Fig. 5) is significantly lower than that of proteinogenic D-AAs (green bars). Including all found D-AAs, proteinogenic and non-proteinogenic, there is a trend that larger peptides with more AAs also contain more D-AAs. Figure 6 plots the number of D-AAs found in natural peptides presented in Tables 2, 3, 4, 5 versus the total number of AAs in the compound to see if there is a trend. 70% of the natural compounds listed in Tables 2, 3, 4, 5 are small peptides containing a maximum of 10 AAs. The linear regression obtained for the relation [n D-AAs] versus [total AAs] is: *number* *of* *D-AA* = 0.42 × *total* *AA* *number* (Fig. 6 green dotted line). The 0.42 slope means that, on average, in D-AA containing natural products, two D-AAs are encountered for each five AAs (Fig. 6). This linear trend has a loose 0.73 regression coefficient with the 30% larger compounds having more weight than the 70% smaller ones. However, an outlier with 48 AAs, polytheonamide **{*125*}** (Table 5), is predicted to contain 19 D-AAs. It contains only 18, a close value. On the other hand, cyclosporine A **{*86*}** is an undecapeptide, it should contain two or even three D-AAs. Only one was found (Table 4). Considering polytheonamide A **{*125*}** as an outlier and excluding it, a quadratic regression provides a slightly better fit of the Fig. 3 points (purple dashed line and regression equation in Fig. 6). The regression coefficient is similar. The quadratic equation predicts that a 20 AA peptide should contain 11 D-amino-acids when the linear fit predicts only 8. Jessineptin **{*20*}** (Table 2) contains 19 AAs in which 13 are D-AAs. The quadratic fit is better than the linear one for large peptides. Neither fit predicts well the number of D-AAs in small peptides. However, a trend is perceptible: peptides with more than 10 AAs will contain several D-AAs.

## Conclusions

The presence of unusual D-amino acids in natural peptide products was first identified by natural product chemists and biochemists. For years, they were the only researchers discovering and identifying an increasing number of D-AA containing natural products. The protocol was to purify a new natural peptide product, then analyze its AA sequence to obtain its formula and then synthesize the all L-AA containing peptide for biological activity. This step was often the one detecting AA chirality when the all L-AA synthesized compound was inactive (e.g. dermorphin **{*129*}**) implying the presence of D-AAs.

The occurrence of D-AAs in natural products having significant biological activities was demonstrated in a wide variety of cases involving monocellular organisms, bacteria, fungi or algae. For a long period of time, D-AAs were not sought in vertebrates that were unquestionably regarded as made exclusively of L-AAs. That may explain why the D-AA occurrence was not reported in organized living forms until recently. The all L-AA dogma was destroyed when D-AAs were found in compounds secreted by invertebrates, vertebrate amphibians and even in one particular mammal: the Australian platypus. Since the presence of D-AAs in more and more natural and biological compounds is detected as the technologies improve, an increasing part of the scientific community has become aware of the need to know the chiral status of AAs in natural products as well as in proteomic studies and obviously in peptide-based pharmaceutical compounds. If D-AAs are sought after, they may be found in many more compounds that what is now known [11]. A powerful method to detect D-AAs in peptide was recently proposed [153].

The function of the known D-containing natural compounds generated by living organisms is to favor the survival and development of the producing entity either by impairing the growth of competing organisms (antibiotics, cytotoxics, siderophores), or by facilitating the entity's expansion (surfactants). These natural products were sought for their bioactivity that could have important applications in medicine, pharmacy and agriculture. It was also demonstrated that free D-AAs were occasionally used by complex eco-systems [152].

Since the standard genetic code encodes only the 19 proteinogenic L-AAs, natural compounds containing D-AAs are mostly produced by NRPS routes. To date, there is no evidence that ribosomes can directly incorporate D-AAs in a peptide chain [152]. However, RiPP post-tranlational modifications of ribosomic all L-AA peptides into D-containing epimeric forms are known and may not be as rare as once thought. With the massive progress made in bioinformatic scanning of the genetic code for a vastly increasing number of living animals and plants, the biosynthetic gene cluster code needed to produce a given peptide or even protein can be calculated and searched within databases in a short time [150]. The software antiSMASH searching for antibiotics and secondary metabolite biosynthetic gene clusters is freely available at https://antismash.secondarymetabolites.org/ [154].

In most cases, the biological activity of the D-containing natural products is completely different of that of the analogous all-L containing stereoisomer. This greatly helps in locating the D-AA(s) in the peptide chain using organic synthesis followed by biological tests. However, there are a few cases where little or no difference in the biological activity was observed between the D- and L-containing analogous natural products. Since these cases may render the D-forms unnoticed, they may be more common than have been reported [10]. The interesting questions that arise for these rare cases are: why does Nature incorporate such D-amino acids? and does resistance to protease degradation play a role in such cases [155]?

## Data Availability

Since this is a review article, there is no new experimentl data that was generated by the authors.

## Footnotes

1 The number in bold and italic refers to the number assigned to the compound in the first column of the tables.

## References

[1] Berg JM, Tymoczko JL, Stryer JM (2007). Biochemistry.

[2] Delvin MD (2011). Textbook of biochemistry with clinical correlations.

[3] Fisher E (1894). Einfluss der Configuration auf die Wirkung der Enzyme (Influence of configuration on the action of enzymes). Berich Deutsch Chem Gesell.

[4] Fisher E (1906). Untersuchungen über aminosäuren, polypeptide und proteïne.

[5] Abraham EP (1957). Biochemistry of some peptides and steroid antibiotics.

[6] Montecucchi PC, De Castiglione R, Piani S, Gozzini L, Erspamer V (1981). Amino acid composition and sequence of Dermorphin, a novel opiate-like peptide from the skin of *Phyllomedusa*
*Sauvagei*. Int J Pept Prot Res.

[7] Jilek A, Kreil G (2008). D-amino acids in animal peptides. Monatsheffe für Chemie.

[8] Du S, Wey M, Armstrong DW (2023). D-amino acids in biological systems. Chirality.

[9] Liu Y, Wu Z, Armstrong DW, Wolosker H, Zheng Y (2023). Detection and analysis of chiral molecules as disease biomarkers. Nat Rev Chem.

[10] Ollivaux C, Soyez D, Toullec JY (2014). Biogenesis of D-amino acid containing peptides/proteins: where, when, and how?. J Peptide Sci.

[11] Mast DH, Checco JW, Sweedler JV (2021). Advancing D-amino acid-containing peptide discovery in the metazoan. BBA Proteins Proteom..

[12] Walsh CT (2004). Polyketide and nonribosomal peptide antibiotics: modularity and versatility. Science.

[13] Walsh CT, O'Briaen RV, Khosla C (2013). Nonproteinogenic amino-acids building blocks for nonribosomal peptide and hybrid polyketide scaffolds. Angew Chem Int Ed.

[14] Wang H, Fewer DP, Holm L, Rouhiainen L, Sivonen K (2014). Atlas of nonribosomal peptide and polyketide biosynthetic pathways reveals common occurrence of nonmodular enzymes. PNAS.

[15] Barron LD, Hecht L, McColl IH, Blanch EW (2004). Raman optical activity comes to age. Mol Phys.

[16] Schlesinger DH. Proteins, traditional methods of sequence determination. In Worsfold P, Townsend A, Poole C (Eds) Encyclopedia of analytical science, 3rd edn., Vol. 8, pp. 352–357. 10.1016/B0-12-369397-7/00497-0

[17] Marfey P (1984). Determination of D-amino acids. II. Use of a bifunctional reagent, 1,5-difluoro-2,4-dinitrobenzene. Carlsberg Res Comm.

[18] Sung YS, Berthod A, Roy D, Armstrong DW (2021). A closer examination of 6-aminoquinolyl-*N*-hydroxysuccinimudyl carbamate amino acid derivation in HPLC with multiple detection modes. Chromatographia.

[19] Stalcup AM (2010). Chiral separations. Ann Rev Anal Chem.

[20] Readel ER, Wey M, Armstrong DW (2021). Rapid and selective separation of amyloid beta from its stereoisomeric point mutations implicated in neurodegenerative Alzheimer disease. Anal Chim Acta.

[21] Du S, Readel ER, Wey M, Armstrong DW (2020). Complete identification of all 20 relevant epimeric peptides in β-amyloid: a new HPLC-MS based analytical strategy for Alzheimer’s research. Chem Commun.

[22] Berthod A, Liu Y, Bagwill C, Armstrong DW (1996). Facile LC enantioresolution of native amino acids and peptides using a teicoplanin chiral stationary phase. J Chromatogr A.

[23] Wimalasinghe R, Breitbach ZS, Lee JT, Armstrong DW (2017). Separation of peptides on superficially porous particles based macrocyclic glycopeptide liquid stationary phases: consideration of fast separations. Anal Bioanal Chem.

[24] Arnstein HRV, Margreiter H (1958). The biosynthesis of penicillin. Biochem J.

[25] Mahariel MA, Essen LO (2009). Nonribosomal peptide synthetases: mechanistic and structural aspects of essential domains. Meth Enzymol.

[26] Raush C, Hoof I, Weber T, Wohlleben W, Huson D (2007). Phylogenetic analysis of condensation domains in NRPS sheds light on their functional evolution. BMC Evolution Biol.

[27] Cheng YQ (2006). Deciphering the biosynthetic codes for the potent anti-SARS-CoV cyclodepsipeptideValinomycin in *Streptomyces*
*tsusimaensis* ATCC 15141. ChemBioChem.

[28] Allard STM, Giraud MF, Naismith JH (2001). Epimerases; structure, function and mechanism. Cell Mol Life Sci.

[29] Arnison PG, Bibb MJ, Bierbaum G (2013). Ribosomally synthesized and post-translationally modified peptide natural products: overview and recommendations for a universal nomenclature. Nat Prod Rep.

[30] Cotter PD, O’Connor PM, Draper LA, Lawton EM, Deegan LH, Hill C, Ross RP (2005). Posttranslational conversion of L-serines to D-alanines is vital for optimal production and activity of the lantibiotic lacticin 3147. Proc Natl Acad Sci USA.

[31] Miao V, Brost R, Chapple J (2006). The lipopeptide antibiotic A54145 biosynthetic gene cluster from *Streptomyces*
*fradiae*. J Ind Microbiol Biotechnol.

[32] Ciferri O, Albertini A, Cassani G (1965). Origin of the sarcosine molecules of actinomycins. Biochem J.

[33] Höltzel A, Schmid DG, Nicholson GJ (2002). Arylomycins A and B, new biaryl-bridged lipopeptide antibiotics produced by Streptomyces sp. Tü 6075. II. Structure elucidation. J Antibiot.

[34] Peypoux F, Pommier MT, Das BC (1984). Structures of bacillomycin D and bacillomycin L peptidolipid antibiotics from *Bacillus*
*subtilis*. J Antibiot.

[35] Ikai Y, Oka H, Hayakawa J (1995). Total structures and antimicrobial activity of bacitracin minor components. J Antibiot.

[36] Franz J, Kazmaier U, Truman AW, Koehnke J (2021). Bottromycins—biosynthesis, synthesis and activity. Nat Prod Rep.

[37] Epperson JD, Ming LJ (2000). Proton NMR studies of Co(ii) complexes of the peptide antibiotic bacitracin and analogues: Insight into structure−activity relationship. Biochemistry.

[38] Wu X, Ballard J, Jiang YW (2005). Structure and biosynthesis of the BT peptide antibiotic from *Brevibacillus*
*texasporus*. Appl Environ Microbiol.

[39] Hojati Z, Milne C, Harvey B (2002). Structure, biosynthetic origin, and engineered biosynthesis of calcium-dependent antibiotics from *Streptomyces*
*coelicolor*. Chem Biol.

[40] Ramesh S, Govender T, Kruger HG, Alberico F, Dela Torre BG (2016). An improved and efficient strategy for the total synthesis of a colistin-like peptide. Tetrahedron Lett.

[41] Baltz RH, Miao V, Wrigley SK (2005). Natural products to drugs: daptomycin and related lipopeptide antibiotics. Nat Prod Rep.

[42] Debono M, Barnhart M, Carrell CB (1987). A21978C, a complex of new acidic peptide antibiotics: isolation, chemistry, and mass spectral structure elucidation. J Antibiot.

[43] Miao V, Coeffet-Legal MF, Brian P (2005). Daptomycin biosynthesis in *Streptomyces*
*roseosporus*: cloning and analysis of the gene cluster and revision of peptide stereochemistry. Microbiology.

[44] Sugawara K, Numata K, Konishi M, Kawaguchi H (1984). Empedopeptin (BMY-28117), a new depsipeptide antibiotic. II. Structure determination. J Antibiot.

[45] Yin X, Zabriskie TM (2006). The enduracidin biosynthetic gene cluster from *Streptomyces*
*fungicidicus*. Microbiology.

[46] Li J, Jensen SE (2008). Nonribosomal biosynthesis of fusaricidins by *Paenibacillus*
*polixyma*
*PKB1* involves direct activation of D-amino acid. Chem Biochem.

[47] Govaerts C, Orwa J, Schepdael AV, Roets E, Hoogmartens J (2001). Structure elucidation of four related substances in gramicidin with liquid chromatography/mass spectrometry. Rapid Com Mass Spec.

[48] Tamaki M, Takimoto M, Sofuku S, Muramatsu I (1983). Synthetic studies on gratisin.II. J Antibiot.

[49] Besson F, Hourdou ML, Michel G (1990). Studies on the biosynthesis of iturin, an antibiotic of *Bacillus*
*subtilis*, and a lipopeptide containing beta-hydroxy fatty acids. Biochim Biophys Acta.

[50] Martin NI, Sprules T, Carpenter MR, Cotter PD, Hill C, Ross RP, Vederas JC (2004). Structural characterization of Lactisin 3147, a two-peptide lantibiotic with synergistic activity. Biochemistry.

[51] Arp J, Götze S, Mukherji R (2018). Synergistic activity of cosecreted natural products from amoebae-associated bacteria. Proc Natl Acad Sci USA.

[52] O'Sullivan J, McCullough JE, Tymiak AA, Kirsch DR, Trejo WH, Principe PA (1988). Lysobactin, a novel antibacterial agent produced by *Lysobacter* sp. I. Taxonomy, isolation and partial characterization. J Antibiot.

[53] Gerard J, Lloyd R, Barsby T, Haden P, Kelly MT, Andersen RJ (1997). Massetolides A-H, antimycobacterial cyclic depsipeptides produced by two pseudomonads isolated from marine habitats. J Nat Prod.

[54] Martin NI, Hu H, Moake MM (2003). Isolation, structural characterization, and properties of Mattacin (Polymyxin M), a cyclic peptide antibiotic produced by *Paenibacillus*
*kobensis*
*M*. J Biol Chem.

[55] Schlusselhuber M, Godard J, Sebban M (2018). Characterization of Milkisin, a novel lipopeptide with antimicrobial properties produced by *pseudomonas*
*sp.* ucma 17988 isolated from bovine raw milk. Front Microbiol.

[56] Sengupta S, Banerjee AB, Bose SK (1971). Gamma-glutamyl and D- or L-peptide linkages in mycobacillin, a cyclic peptide antibiotic. Biochem J.

[57] Okumura Y, Sakamoto M, Takei T, Ishikura T, Fukagawa Y (1979). Controlled biosynthesis of neoviridogriseins, new homologues of viridogrisein. IV. In vitro synergism between neoviridogrisein II and the antibiotics of the mikamycin A group. J Antibiot.

[58] Queener SW (1990). Molecular biology of penicillin and cephalosporin biosynthesis. Antimicrob Agents Chemother.

[59] Shoji J, Hinoo H, Katayama T, Nakagawa Y, Ikenishi Y, Iwatani K, Yoshida T (1992). Structures of new peptide antibiotics, plusbacins A1–A4 and B1–B4. J Antibiot.

[60] Govaerts C, Orwa J, Schepdael AV, Roets E, Hoogmartens J (2002). Characterization of polypeptide antibiotics of the polymyxin series by LC-ESI-ion trap tandem MS. J Pept Sci.

[61] Bhattacharjya S, David SA, Mathan VI, Balaram P (1997). Polymyxin B nonapeptide: conformation in water and in the lipopolysaccharide-bound state determined by 2D-NMR and molecular dynamics. Biopol.

[62] de Crécy-Lagard V, Saurin W, Thibaut D, Gil P, Naudin L, Crouzet J, Blanc V (1997). Streptogramin B biosynthesis in *Streptomyces*
*pristinaespiralis* and *Streptomyces*
*virginiae*: molecular characterization of the last structural peptide synthetase gene. Antimicrob Agents Chemother.

[63] Martin DG, Mizsak SA, Biles C, Stewart JC, Meulman PA (1975). Structure of quinomycin antibiotics. J Antibiot.

[64] McCafferty DG, Cudic P, Frankel BA, Barkallah S, Kruger RG, Li W (2002). Chemistry and biology of the ramoplanin family of peptide antibiotics. Biopolymers.

[65] Harris CM, Kibby JJ, Fehlner JR, Raabe JR, Barber TA, Harris TM (1979). Amino acid constituents of Ristocetin A. J Am Chem Soc.

[66] Segre A, Bachmann RC, Ballio A (1989). The structure of syringomycins A1, E and G. FEBS Lett.

[67] Grgurina I, Mariotti F (1999). Biosynthetic origin of syringomycin and syringopeptin 22, toxic secondary metabolites of the phytopathogenic bacterium *Pseudomonas*
*syringae* pv.. FEBS Lett.

[68] Carpaneto A, Dalla Serra M, Menestrina G, Fogliano V, Gambale F (2002). The phytotoxic lipodepsipeptide Syringopeptin 25A from *Pseudomonas*
*syringae*
*pv*
*syringae* forms ion channels in sugar beet vacuoles. J Membr Biol.

[69] Berthod A. Macrocyclic glycopeptides chiral stationary phases. In Reference Collection in Chemistry, Molecular Sciences and Chemical Engineering, 2022, Elsevier, Amsterdam. 10.1016/B978-0-32-390644-9.00004-4.

[70] Bassarello C, Lazzaroni S, Bifulco G (2004). Tolaasins A−E, five new lipodepsipeptides produced by *Pseudomonas*
*tolaasii*. J Nat Prod.

[71] Otsuka H, Shoji J, Kawano K, Kyogoku Y (1976). Structure confirmation of triostin a by 1H and 13C magnetic resonance. J Antibiot.

[72] Mootz HD, Marahiel MA (1997). The tyrocidine biosynthesis operon of *Bacillus*
*brevis*: complete nucleotide sequence and biochemical characterization of functional internal adenylation domains. J Bacteriol.

[73] Cheng YQ (2006). Deciphering the biosynthetic codes for the potent anti-SARS-CoV cyclodepsipeptide valinomycin in *Streptomyces*
*tsusimaensis* ATCC 15141. ChemBioChem.

[74] Zhou Q, Grundmann F, Kaiser M (2013). Structure and biosynthesis of Xenoamicins from *Entomopathogenic*
*xenorhabdus*. Chem Eur J.

[75] Demange P, Bateman A, Dell A, Abdallah MA (1988). Structure of azotobactin D, a siderophore of *Azotobacter*
*vinelandii* strain D (CCM 289). Biochemistry.

[76] Persmark M, Expert D, Neilands JB (1989). Isolation, characterization, and synthesis of chrysobactin, a compound with siderophore activity from *Erwinia*
*chrysanthemi*. J Biol Chem.

[77] Risse D, Beiderbeck H, Taraz K, Budzikiewicz H, Gustine D (1998). Corrugatin, a lipopeptide siderophore from *Pseudomonas*
*corrugata*. Zeitschrift für Naturforschung C.

[78] Xu G, Martinez JS, Groves JT, Butler A (2002). Membrane affinity of the amphiphilic Marinobactin siderophores. J Am Chem Soc.

[79] Persmark M, Frejd T, Mattiasson B (1990). Purification, characterization, and structure of pseudobactin 589 A, a siderophore from a plant growth promoting *Pseudomonas*. Biochemistry.

[80] Georgias H, Taraz K, Budzikiewicz H, Geoffroy V, Meyer J-M (1999). The structure of the pyoverdin from *Pseudomonas*
*fluorescens* 1.3. Structural and biological relationships of pyoverdins from different strains. Zeitschrift für Naturforschung C..

[81] Fernández DU, Fuchs R, Schäfer M, Budzikiewicz H, Meyer JM (2003). The pyoverdin of *Pseudomonas*
*fluorescens* G173, a novel structural type accompanied by unexpected natural derivatives of the corresponding ferribactin. Zeitschrift fur Naturforschung C.

[82] Grim KP, Francisco BS, Radin JN (2017). The metallophore Staphylopine enables *Staphylococcus*
*aureus* to compete with the host for Zinc and overcome nutritional immunity. MBio.

[83] Gwose I, Taraz K (1992). Pyoverdins from *Pseudomonas*
*putida*. Zeitschrift fur Naturforschung C.

[84] Schalk IJ, Rigouin C, Godet J (2020). An overview of siderophore biosynthesis among fluorescent *Pseudomonas* and new insights into their complex cellular organization. Env Microbiol.

[85] Sørensen D, Nielsen TH, Christophersen C, Sørensen J, Gajhede M (2001). Cyclic lipoundecapeptide amphisin from *Pseudomonas*
*sp*. strain DSS73. Acta Crystal C Crystal Struct Comm..

[86] Fukui Y, Narita K, Dan S (2014). Total synthesis of burkholdacs A and B and 5,6,20-tri-epi-burkholdac A. Eur J Med Chem.

[87] Ehling-Schulz M, Fricker M, Grallert H, Rieck P, Wagner M, Scherer S (2006). Cereulide synthetase gene cluster from emetic *Bacillus*
*cereus*: structure and location on a mega virulence plasmid related to *Bacillus*
*anthracis* toxin plasmid pXO1. BMC Microbiol.

[88] Vanittanakom N, Loeffler W, Koch U, Jung G (1986). Fengycin—a novel antifungal lipopeptide antibiotic produced by Bacillus subtilis F-29-3. J Antibiot.

[89] Ballio A, Bossa F, Camoni L (1996). Structure of fuscopeptins, phytotoxic metabolites of *Pseudomonas*
*fuscovaginae*. FEBS Lett.

[90] Pettit GR, Tan R, Melody N (1999). Antineoplastic agents. Part 409: isolation and structure of montanastatin from a terrestrial actinomycete. Bioorg Med Chem.

[91] Gross H, Stockwell VO, Henkels MD, Nowak-Thompson B, Loper JE, Gerwick WH (2007). The genomisotopic approach: a systematic method to isolate products of orphan biosynthetic gene clusters. Chem Biol.

[92] Quail JW, Ismail N, Pedras MS, Boyetchko SM (2002). Pseudophomins A and B, a class of cyclic lipodepsipeptides isolated from a *Pseudomonas* species. Acta Crystal C, Crystal Struct Comm..

[93] Morikawa M, Daido H, Takao T, Murata S, Shimonishi Y, Imanaka S (1993). A new lipopeptide biosurfactant produced by *Arthrobacter*
*sp*. strain MIS38. J Bacteriol.

[94] Béchet M, Caradec T, Hussein W (2012). Structure, biosynthesis, and properties of kurstakins, nonribosomal lipopeptides from *Bacillus* spp. Appl Microbiol Biotechnol.

[95] Matsuyama T, Kaneda K, Nakagawa Y, Isa K, Hara-Hotta H, Yano I (1992). A novel extracellular cyclic lipopeptide which promotes flagellum-dependent and -independent spreading growth of *Serratia*
*marcescens*. J Bacteriol.

[96] Henriksen A, Anthoni U, Nielsen TH, Sorensen J, Christophersen C, Gajhede M (2000). Cyclic lipoundecapeptide tensin from *Pseudomonas*
*fluorescens* strain 96.578. Acta Crystal C Crystal Struct Comm..

[97] Laycock MV, Hildebrand PD, Thibault P, Walter JA, Wright JLC (1991). Viscosin, a potent peptidolipid biosurfactant and phytopathogenic mediator produced by a pectolytic strain of *Pseudomonas*
*fluorescens*. J Agric Food Chem.

[98] Martinez JS, Carter-Franklin JN, Mann EL, Martin JD, Haygood MG, Butler A (2003). Structure and membrane affinity of a suite of amphiphilic siderophores produced by a marine bacterium. Proc Nat Acad Sci.

[99] Itou Y, Suzuki S, Ishida K, Murakami M (1999). Anabaenopeptins G and H, potent carboxypeptidase A inhibitors from the cyanobacterium *Oscillatoria*
*agardhii* (NIES-595). Bioorg Med Chem Lett.

[100] Williams DE, Craig M, Holmes CFB, Andersen RJ (1996). Ferintoic acids A and B, new cyclic hexapeptides from the freshwater cyanobacterium *Microcystis*
*aeruginosa*. J Nat Prod.

[101] Gao J, Hamann MT (2011). Chemistry and biology of Kahalalides. Chem Rev.

[102] Kan Y, Fujita T, Sakamoto B, Hokama Y, Nagai H, Kahalalide K (1999). A new cyclic depsipeptide from the hawaiian green alga bryopsis species. J Nat Prod.

[103] Namikoshi M, Rinehart KL, Sakai R (1992). Identification of 12 hepatotoxins from a Homer Lake bloom of the cyanobacteria *Microcystis*
*aeruginosa*, *Microcystis*
*viridis*, and *Microcystis*
*wesenbergii*: nine new microcystins. J Org Chem.

[104] Sivonen K, Namikoshi M, Evans WR, Färdig M, Carmichael WW, Rinehart KL (1992). Three new microcystins, cyclic heptapeptide hepatotoxins, from *Nostoc* sp. strain 152. Chem Res Toxicol.

[105] Mazur-Marzec H, Meriluoto J, Pliński M, Szafranek J (2006). Characterization of nodularin variants in *Nodularia*
*spumigena* from the Baltic Sea using LC/MS/MS. Rapid Commun Mass Spectrom.

[106] Hanessian S, Tremblay M, Petersen JFW (2004). The N-Acyloxyiminium ion aza-prins route to octahydroindoles: total synthesis and structural confirmation of the antithrombotic marine natural product Oscillarin. J Am Chem Soc.

[107] Meriluoto JA, Sandström A, Eriksson JE, Remaud G, Grey Craig A, Chattopadhyaya J (1989). Structure and toxicity of a peptide hepatotoxin from the cyanobacterium *Oscillatoria*
*agardhii*. Toxicon.

[108] Singh SB, Zink DL, Liesch JM (2002). Structure and chemistry of Apicidins, a class of novel cyclic tetrapeptides without a terminal α-keto epoxide as inhibitors of histone deacetylase with potent antiprotozoal activities. J Org Chem.

[109] Kuzma M, Jegorov A, Kačer P, Havlíček V (2001). Sequencing of new beauverolides by HPLC/MS. J Mass Spectrom.

[110] Closse A, Huguenin R (1974). Isolierung und strukturaufklärung von Chlamydocin. Helv Chim Acta.

[111] Lawen A, Zocher R (1990). Cyclosporin synthetase. The most complex peptide synthesizing multienzyme polypeptide so far described. J Biol Chem.

[112] Scott-Craig JS, Panaccione DG, Pocard JA, Walton JD (1992). The cyclic peptide synthetase catalyzing HC-toxin production in the filamentous fungus *Cochliobolus*
*carbonum* is encoded by a 15.7-kilobase open reading frame. J Biol Chem.

[113] Walton JD (2005). HC-toxin. Phytochemistry.

[114] Baute R, Deffieux G, Merlet D, Baute MA, Neveu A (1981). New insecticidal cyclodepsipeptides from the fungus *Isaria*
*feline*. II. Structure elucidation of isariins B. C and D. J Antibiot..

[115] Sugawara F, Kim KW, Uzawa J, Yoshida S, Takahashi N, Curtis RW (1990). Structure of malformin A2, reinvestigation of phytotoxic metabolites produced by *aspergillus*
*niger*. Tetrahedron Lett.

[116] Kim KW, Sugawara F, Yoshida S, Murofushi N, Takahashi N, Curtis RW (1993). Structure of malformin B, a phytotoxic metabolite produced by *Aspergillus*
*niger*. Biosci Biotechnol Biochem.

[117] Anderegg RJ, Biemann K, Büchi G, Cushman M (1976). Malformin C, a new metabolite of *Aspergillus*
*niger*. J Am Chem Soc.

[118] Bodanszky M, Bednarek MA, Yiotakis AE, Curtis RW (1982). Allomalformin. Int J Pept Protein Res.

[119] Nishikawa M, Ogawa K (2004). Occurrence of D-histidine residues in antimicrobial poly(arg-his) conferring resistance to enzymatic hydrolysis. FEMS Microb Lett.

[120] Goetz G, Nakao Y, Scheuer PJ (1997). Two acyclic Kahalalides from the Sacoglossan mollusk *Elysia*
*rufescens*. J Nat Prod.

[121] Tilvi S, Naik CG (2007). Tandem mass spectrometry of kahalalides: identification of two new cyclic depsipeptides, kahalalide R and S from *Elysia*
*grandifolia*. J Mass Spectrom.

[122] Rao KV, Na M, Cook JC, Peng J, Matsumoto R, Hamann MT (2008). Kahalalides V-Y isolated from a Hawaiian collection of the sacoglossan mollusk *Elysia*
*rufescens*. J Nat Prod.

[123] Fernández R, Rodriguez J, Quiñoa E (1996). Onchidin B: a new cyclodepsipeptide from the mollusc *Onchidium*
*sp.*. J Am Chem Soc.

[124] Katamani Y, Minakata H, Kenny PTM (1989). Achatin I, an endogenous neuroexitatory tetrapeptide for *Achata*
*fulica* containing a D-amino acid residue. Biochem Biophys Res Comm.

[125] Roy MC, Ohtani II, Tanaka J, Higa T, Satari R (1999). Barangamide A, a new cyclic peptide from the Indonesian sponge *Theonella*
*swinhoei*. Tetrahedron Lett.

[126] Trevisi L, Bova G, Cargnelli G (2000). Callipeltin A, a cyclic depsipeptide inhibitor of the cardiac sodium-calcium exchanger and positive inotropic agent. Biochem Biophys Res Commun.

[127] Calimsiz S, Morales Ramos AI, Lipton MA (2006). Solid-phase synthesis and configurational reassigment of callipeltin E. Implications for the structures of callipeltins A and B. J Org Chem.

[128] D’Auria MV, Sepe V, D’Orsi R, Bellotta F, Debitus C, Zampella A (2007). Isolation and structural elucidation of callipeltins J–M: antifungal peptides from the marine sponge *Latrunculia* sp.. Tetrahedron.

[129] Bonnington LS, Tabaka J, Higa T, Kimura J, Yoshimura Y, Nakao Y, Scheuer PJ (1997). Cupolamide A: a cytotoxic cyclic heptapeptide from two samples of the sponge *Theonella*
*cupola*. J Org Chem.

[130] Nakao Y, Matsunaga S, Fusetani N (1995). Three more cyclotheonamides, C, D, and E, potent thrombin inhibitors from the marine sponge *Theonella*
*swinhoei*. Biorg Med Chem.

[131] Sato K, Horibe K, Amano K (2001). Membrane permeabilization induced by discodermin A, a novel marine bioactive peptide. Toxicon.

[132] Li HY, Matsunaga S, Fusetani N (1995). Halicylindramides A-C, antifungal and cytotoxic depsipeptides from the marine sponge *Halichondria*
*cylindrata*. J Med Chem.

[133] Kobayashi JI, Itagaki F, Shigemori I, Takao T, Shimonishi Y (1995). Keramamides E, G, H, and J, new cyclic peptides containing an oxazole or a thiazole ring from a *Theonella* sponge. Tetrahedron.

[134] Araki T, Matsunaga S, Fusetani N (2005). Koshikamide A2, a cytotoxic linear undecapeptide from a marine sponge of *Theonella*
*sp*. Biosci Biotech Biochem.

[135] Qureshi A, Colin PL, Faulkner DJ (2000). Microsclerodermins F-I, antitumor and antifungal cyclic peptides from the lithistid sponge *Microscleroderma*
*sp*. Tetrahedron.

[136] Rashid MA, Gustafson KR, Cartner LK, Shigematsu N, Pannell LK, Boyd MR (2001). Microspinosamide, a new HIV-inhibitory cyclic depsipeptide from the marine sponge *Sidonops*
*microspinosa*. J Nat Prod.

[137] de Silva ED, Williams DE, Andersen RJ, Klix H, Holmes FB, Allen TM (1992). Motuporin, A potent protein phosphatase inhibitor isolated from the papua new guinea sponge *Theonella*
*swinhoei*
*Gray*. Tetrahedron Lett.

[138] Schmidt EW, Harper MK, Faulkner DJ (1997). Mozamides A and B, cyclic peptides from a *Theonellid* sponge from Mozambique. J Nat Prod.

[139] Oku N, Gustafson KR, Cartner LK (2004). Neamphamide A, a new HIV-inhibitory depsipeptide from the Papua New Guinea marine sponge *Neamphius*
*huxleyi*. J Nat Prod.

[140] Capon RJ, Ford J, Lacey E, Gill JH, Heiland K, Friedel T (2002). Phoriospongin A and B: two new nematocidal depsipeptides from the Australian marine sponges *Phoriospongia* sp. and *Callyspongia*
*bilamellata*. J Nat Prod.

[141] Gulavita NK, Gunasekera SP, Pomponi SA, Robinson EV (1992). Polydiscamide A: a new bioactive depsipeptide from the marine sponge *Discodermia*
*sp*. J Org Chem.

[142] Feng Y, Carroll AR, Pass DM, Archbold JK, Avery VM, Quinn RJ (2008). Polydiscamides B-D from a marine sponge *Ircinia* sp. as potent human sensory neuron-specific G protein coupled receptor agonists. J Nat Prod.

[143] Hamada T, Matsunaga S, Yano G, Fusetani N (2005). Polytheonamides A and B, highly cytotoxic, linear polypeptides with unprecedented structural features, from the marine sponge, *Theonella*
*swinhoei*. J Am Chem Soc.

[144] Nakao Y, Masuda A, Matsunaga S, Fusetani N (1999). Pseudotheonamides, serine protease inhibitors from the marine sponge *Theonella*
*swinhoei*
*1*. J Am Chem Soc.

[145] Roy MC, Ohtani II, Ichiba T, Tanaka J, Satari R, Higa T (2000). New cyclic peptides from the Indonesian sponge *Theonella*
*swinhoei*. Tetrahedron.

[146] Li S, Dumdei EJ, Blunt JW, Munro MHG, Robinson WT, Pannell LK (1998). Theonellapeptolide IIIe, a new cyclic peptolide from the New Zealand deep water sponge, *Lamellomorpha*
*strongylata*. J Nat Prod.

[147] Keppel-Hesselink JM, Schatman ME (2018). Rediscovery of old drug: the forgotten case of Dermorphin for postoperative pain and palliation. J Pain Res.

[148] Misicka A, Lipkowski AW, Horvath R, Davis P, Kramer TH, Hruby VJ (1992). Topographical requirements for delta opioid ligands: common structural features of dermenkephalin and deltorphin. Life Sci.

[149] Mignogna G, Simmaco M, Kreil G, Barra D (1993). Antibacterial and haemolytic peptides containing D-alloisoleucine from the skin of *Bombina*
*variegata*. EMBO J.

[150] Torres AM, Tsampazi C, Geraghty DP, Bansal PS, Alewood PF (2005). D-Amino acid residue in a defensin-like peptide from platypus venom: effect on structure and chromatographic properties. Biochem J.

[151] Freeman MF, Vagstadt AL, Piel J (2016). Polytheonamide biosynthesis showcasing the metabolic potential of sponge-associated uncultivated *Entotheonella* bacteria. Curr Op Chem Biol.

[152] Aliashkevich A, Alvarez L, Cava F (2018). New insights into the mechanisms and biological roles of D-amino acids in complex eco-systems. Front Microbiol.

[153] Livnat I, Tai HC, Jansson ET, Bai L, Romanova EV, Chen TT, Liu DD, Weiss KR, Jing J, Sweedler JV (2016). A D-amino acid containing neuropeptide discovery funnel. Anal Chem.

[154] Blin K, Shaw S, Augustijn HE (2023). AntiSMASH 7.0: new and improved predictions for detection, regulation, chemical structures, and visualization. Nucl Acids Res..

[155] Sung YS, Khvalbota L, Dhaubhadel U, Spanik I, Armstrong DW (2023). Teicoplanin aglycone media and carboxypeptidase Y: tools for finding low-abundance D-amino acids and epimeric peptides. Chirality.

